# Genome-wide and molecular evolution analyses of the phospholipase D gene family in Poplar and Grape

**DOI:** 10.1186/1471-2229-10-117

**Published:** 2010-06-18

**Authors:** Qi Liu, Chiyu Zhang, Yongping Yang, Xiangyang Hu

**Affiliations:** 1 Kunming Institute of Botany, Institute of Tibetan Plateau Research at Kunming, Chinese Academy of sciences, Kunming, Yunnan, 650204, China; 2 Institute of Life Sciences, Jiangsu University, Zhenjiang, Jiangsu 212013, China

## Abstract

**Background:**

The Phospholipase D (PLD) family plays an important role in the regulation of cellular processes in plants, including abscisic acid signaling, programmed cell death, root hair patterning, root growth, freezing tolerance and other stress responses. PLD genes constitute an important gene family in higher plants. However, until now our knowledge concerning the PLD gene family members and their evolutionary relationship in woody plants such as Poplar and Grape has been limited.

**Results:**

In this study, we have provided a genome-wide analysis of the PLD gene family in Poplar and Grape. Eighteen and eleven members of the PLD gene family were identified in Poplar and Grape respectively. Phylogenetic and gene structure analyses showed that the PLD gene family can be divided into 6 subgroups: α, β/γ, δ, ε, ζ, and φ, and that the 6 PLD subgroups originated from 4 original ancestors through a series of gene duplications. Interestingly, the majority of the PLD genes from both Poplar (76.5%, 13/17) and Grape (90.9%, 10/11) clustered closely together in the phylogenetic tree to the extent that their evolutionary relationship appears more tightly linked to each other, at least in terms of the PLD gene family, than it does to either Arabidopsis or rice. Five pairs of duplicated PLD genes were identified in Poplar, more than those in Grape, suggesting that frequent gene duplications occurred after these species diverged, resulting in a rapid expansion of the PLD gene family in Poplar. The majority of the gene duplications in Poplar were caused by segmental duplication and were distinct from those in Arabidopsis, rice and Grape. Additionally, the gene duplications in Poplar were estimated to have occurred from 11.31 to 13.76 million years ago, which are later than those that occurred in the other three plant species. Adaptive evolution analysis showed that positive selection contributed to the evolution of the PXPH- and SP-PLDs, whereas purifying selection has driven the evolution of C2-PLDs that contain a C2 domain in their N-terminal. Analyses have shown that the C2-PLDs generally contain 23 motifs, more than 17 motifs in PXPH-PLDs that contain PX and PH domains in N-terminal. Among these identified motifs, eight, (6, 8, 5, 4, 3, 14, 1 and 19) were shared by both the C2- and PXPH-PLD subfamilies, implying that they may be necessary for PLD function. Five of these shared motifs are located in the central region of the proteins, thus strongly suggesting that this region containing a HKD domain (named after three conserved H, K and D residues) plays a key role in the lipase activity of the PLDs.

**Conclusion:**

As a first step towards genome wide analyses of the PLD genes in woody plants, our results provide valuable information for increasing our understanding of the function and evolution of the PLD gene family in higher plants.

## Background

Plants are exposed to widely varying environmental conditions and because of their sessile nature they can only survive and thrive by adapting to the changes in their surroundings. Thus, higher plants have the ability to adapt to periods of stress by employing specific responses underpinned by defined modifications of their cellular processes. Phospholipase D (PLD) plays an important role in the regulation of diverse cellular processes in plants, including abscisic acid signaling, programmed cell death, root hair patterning, root growth, freezing tolerance and other stress responses [1]. PLD hydrolyzes phospholipids into a head group alcohol and phosphatidic acid (PA), which is an important intracellular messenger in plants, microorganisms and mammals [2].

The gene encoding PLD was first identified in plants more than 50 years ago [3], but did not receive detailed attention until the 1980s [4,5]. Multiple PLD genes encoding isoforms that could be classified into different subgroups with distinct biochemical, regulatory and catalytic properties have now been identified. Six Arabidopsis PLDs (α, β, γ, δ, ε and ζ) have been characterized molecularly and biochemically and can be differentiated depending on their requirements and/or affinities for Ca^2+^, phosphatidylinositol 4,5-bisphosphate (PIP_2_) and free fatty acids [6,7]. The predominant isoenzyme is the α-type PLD, which can be detected in both the leaves and seeds of plants and is responsible for the majority of the baseline PLD activity found therein. PLDα does not require phosphoinositides for its activity when assayed in the presence of mM levels of Ca^2+ ^ions. It exhibits optimum activity at pH values between 5 and 6 and at high, non-physiological Ca^2+ ^concentrations between 30 and 100 mM [8,9]. In contrast, the β, γ, δ and ε PLD isoenzymes from Arabidopsis show their highest activity at μM Ca^2+ ^concentrations and require the presence of PIP_2 _to be fully active [10]. The activity of plant PLDζ appears to occur independently of Ca^2+ ^ions, but requires PIP_2 _to selectively hydrolyze phosphatidylcholine. In rice, an additional isoenzyme, PLDφ, has been identified but poorly characterized as of yet [11]. The PLD gene family encodes proteins with a number of cellular functions. For example, it has been suggested that PLDβ is involved in the regulation of seed germination and may act as a negative regulator of defence responses and disease resistance in rice [11,12], whereas PLDδ has been shown to play an important role in drought-induced hydrogen peroxide synthesis, responses to freezing and UV irradiation, and in the reorganization of microtubules at plasma membrane [1,13].

Despite these apparent differences in their biochemical functions, all the eukaryotic PLDs share the presence of an N-terminal phospholipid-binding region and two highly conserved C-terminal domains where two catalytic HxKxxxxD (HKD) motifs interact to promote the lipase activity [14,15]. The plant PLD family can also be divided into two further subfamilies (C2 and PXPH) based on the composition of their N-terminal phospholipid-binding domains. The C2-PLD subfamily comprises PLDs containing a C2 domain in their N-termini, while the N-termini of those of the PXPH-PLD subfamily contain both a phox homology (PX) domain and a pleckstrin homology (PH) domain. The C2, PX and PH domains have been implicated in protein-protein interactions, but perhaps their best described function involves their ability to modulate membrane targeting of proteins. The C2 domain of the C2-PLDs mediates the localization of soluble proteins to membranes by binding phospholipids in a Ca^2+ ^dependent manner [16], while the PX and PH domains of the PXPH-PLDs have been shown to mediate membrane targeting and are closely linked to polyphosphoinositide signalling [17]. The C2-PLDs only exist in plants, whereas the PXPH-PLDs exist both in plants and other organisms such as *Caenorhabditis elegans *and *Homo sapiens*. Presumably, the genes encoding the C2-PLDs and their progenitors have been lost from the evolutionary lineages leading to animals and fungi [18]. Furthermore, one additional small PLD subfamily (SP-PLDs) exists in which members comprise PLDs possessing an N-terminal signal peptide in place of the usual C2 or PXPH domains and the resulting specific cellular localizations may relate to their particular physiological functions in modulating plant growth, development and defence [11]. The isoforms α, β, γ, δ, and ε are C2-PLDs, the ζ isoform is a PXPH-PLDs and the φ isoform is a SP-PLDs.

The PLD gene family had been well studied in Arabidopsis and rice. However, there is far less information about this family for woody plant species such as Poplar and Grape. The recent provision of draft genome sequences for Poplar and Grape offered the opportunity to investigate the PLD gene family in these species. In this study, we first identified the PLD gene family members in Poplar and Grape and then performed detailed evolutionary analyses of these identified genes in comparison with those existing in Arabidopsis and rice.

## Results and Discussion

### PLD gene family in Poplar and Grape

In order to identify members of the PLD gene family in Poplar and Grape, the corresponding sequence information from Arabidopsis was used to perform multiple searches of the relevant DNA databases using the blast and tblastn algorithms, keyword searches and protein domain searches. The Poplar and Grape sequences returned by such searches were confirmed as encoding PLDs by using the programs PFAM and SMART. Following this strategy, we identified 18 genes encoding PLDs in Poplar (including a pseudogene) (Table 1) and 11 PLD genes in Grape (Table 2). These numbers where similar to the number of PLD genes present in the rice (17 PLD genes) and Arabidopsis (12 PLD genes) genomes. Since there was no standard annotation assigned to these newly identified genes, we assigned each of them an identity based on the order of their location on each of either the Poplar or Grape chromosomes.

**Table 1:** PLD genes identified in Poplar

Gene name	NCBI geneID	JGI Id	Genomic position	Gene model	Protein length	EST number	Gene family
PtPLD1	7490921	1067075	LG_I:8392741,8398030(-)	estExt_Genewise1Plus.C_LG_I2870	849	8	C2
PtPLD2	7475119	829577	LG_I:14825540..14830277(-)	estExt_fgenesh4_pm.C_LG_I0571	808	71	C2
PtPLD3	7496896	550827	LG_II:921630..926824(-)	eugene3.00020142	794	0	C2
PtPLD4	7494030	755219	LG_II:11528682,11536278(+)	fgenesh4_pg.C_LG_II001399	1100	5	C2
PtPLD5	7480751	853026	LG_III:539882,549625(-)	e_gw1.III.2315.1	853	0	C2
PtPLD6	7485622	559891	LG_V:17096700..17101855(+)	eugene3.00051506	836	4	C2
PtPLD7	7468635	763496	LG_VI:17171730,17179050(+)	fgenesh4_pg.C_LG_VI001806	791	2	C2
PtPLD8	7477219	833366	LG_X:554905,566018(-)	estExt_fgenesh4_pm.C_LG_X0035	1120	9	PXPH
PtPLD9	7478921	1096093	LG_XIII:888970,894947(-)	estExt_Genewise1Plus.C_LG_XIII0313	1111	2	PXPH
PtPLD10	7496922	730956	LG_XIV:1822618,1829297(+)	estExt_Genewise1_v1.C_LG_XIV0962	798	6	C2
PtPLD11	Not found	779129	LG_XVIII:6311823,6314468(-)	fgenesh4_pg.c_lg_xviii000497	516	3	SP
PtPLD12	7470100	578949	LG_XVIII:12590250,12593859(-)	eugene3.00181173	808	0	C2
PtPLD13	7486135	810176	scaffold_44:490710..496337(-)	fgenesh4_pm.C_scaffold_44000016	808	18	C2
PtPLD14	7486144	781949	scaffold_44:928367..930598(+)	fgenesh4_pg.C_scaffold_44000079	759	1	C2
PtPLD15	7483584	593768	scaffold_57:67007..73365(+)	eugene3.00570012	881	12	C2
PtPLD16	7480531	827396	scaffold_77:955228..967889(-)	estExt_fgenesh4_pg.C_770076	1096	6	PXPH
PtPLD17	7486437	811801	scaffold_181:39105..44450(+)	fgenesh4_pm.C_scaffold_181000001	859	3	C2
PtPLD18*	7485476	417354	LG_VI:4395633..4397434(-)	gw1.VI.1727.1		0	C2

*pseudogene

**Table 2:** PLD genes identified in Grape

Gene name	Gene ID	Locus name	Genomic position	Protein length	EST number	Gene family
VvPLD1	100252995	GSVIVT00001073001	chr2:4092311..4100706(-)	839	1	C2
VvPLD2	100257647	GSVIVT00032653001	chr4:4887871..4891009(+)	788	0	C2
VvPLD3	100242220	GSVIVT00032779001	chr4:6584390..6591687(-)	517	6	SP
VvPLD4	100261987	GSVIVT00033053001	chr5:461312..479667(+)	1073	6	PXPH
VvPLD5	100256679	GSVIVT00034191001	chr9:2139880..2146589(-)	755	0	C2
VvPLD6	100232928	GSVIVT00000347001	chr9:6671424..6677951(+)	812	98	C2
VvPLD7	100261040	Not found	chr11:4654107..4656834(+)	817	0	C2
VvPLD8	100259366	Not found	chr11:4658773..4665399(+)	813	5	C2
VvPLD9	100261019	GSVIVT00035483001	chr12:17242633..17268429(-)	856	0	C2
VvPLD10	100250490	GSVIVT00026323001	chr15:5881393..5883696 (-)	768	11	C2
VvPLD11	100241982	Not found	chr18: 1509290..1517022(+)	840	2	C2

Based on the presence of C2, PX and PH motifs within their N-terminal domains, all the PLD family members in Poplar and Grape were assigned to two main subgroups, C2-PLDs and PXPH-PLDs. Additionally, one gene encoding an SP-PLD with an N-terminal signal peptide replacing the C2, PX and PH domains was identified for each of these species. Corresponding SP-PLD genes were also found in other species, including *Caenorhabditis elegans *(CAE72017, NP_504824), *Dictyostelium discoideum *(XP_637114), *Homo sapiens *(AAH00553, AAH15003) and rice (Os06g44060).

### Chromosomal location of PLD genes on Poplar and Grape genomes

**Figure 1:**
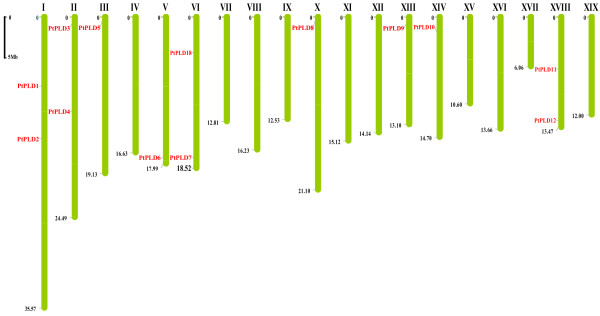
**Positions of PLD gene family members on the Poplar chromosomes**. Scale represents a 5 Mb chromosomal distance. Five PLD genes (PtPLD13, PtPLD14, PtPLD15, PtPLD16 and PtPLD17) reside on unassembled scaffolds.

**Figure 2:**
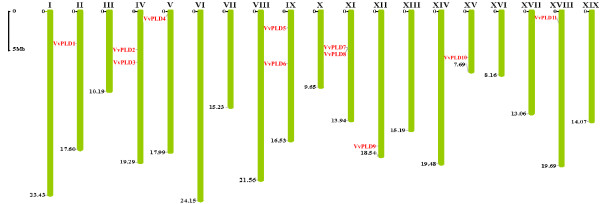
**Positions of PLD gene family members on the Grape chromosomes**. Scale represents a 5 Mb chromosomal distance.

Chromosomal location analyses showed that PLD genes of Poplar and Grape were dispersed throughout the respective genomes. Five Poplar PLD genes were localized to unassembled genomic sequence scaffolds and thus were not mapped to any particular chromosome. In Poplar, chromosomes I, II, VI and XVIII were found to possess two PLD genes each, and each of chromosomes III, V, X, XIII and XIV to possess a single PLD gene (Figure 1). For Grape, 11 PLD genes were found to be present on 8 of the 19 chromosomes; chromosomes II, V, XII, XV and XVIII were all found to possess one PLD gene each, whereas chromosome IV, IX and XI possessed two PLD genes each (Figure 2).

### Phylogenetic relationships of PLD gene family in Poplar and Grape

**Figure 3:**
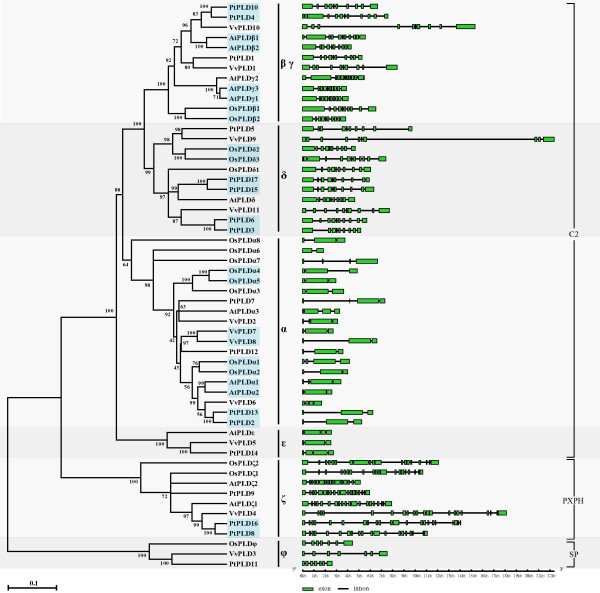
**Phylogenetic analysis and schematic diagram for intron/exon gene structures of PLD genes in Arabidopsis, rice, Poplar and Grape**. The Phylogenetic tree was constructed based on a complete protein sequence alignment of PLDs in the four higher plants by the neighbor-joining method with bootstrapping analysis (1000 replicates). The numbers beside the branches indicate the bootstrap values that support the adjacent node. The green boxes and gray lines in the gene structure diagram represent exons and introns, respectively. Gene models are drawn to scale as indicated on bottom. The gene pairs marked by the blue box represent the 13 paralogous gene pairs.

In order to classify the PLD genes identified for Poplar and Grape and investigate their evolutionary relationships, their derived protein sequences and those of Arabidopsis and rice [6,11] were subjected to phylogenetic analyses. One rice PLD gene, OsPLDκ (Os02g02790), was excluded from the analysis since it appeared to encode a protein missing one HKD domain at its C-terminus, indicating that this gene may be a pseudogene or constitute either a sequencing or assembly error. After excluding other cases of such pseudogenes or incorrectly assembled genes, a total of 56 PLD genes were used in the analyses, 17 from Poplar (excluding the pseudogene PtPLD18), 11 from Grape, 12 from Arabidopsis and 16 from rice [6,11] (Figure 3). A phylogenetic tree based on protein sequences was constructed using the neighbor-joining (NJ) method with p-distance and complete deletion option. For statistical reliability, we conducted bootstrap analysis with 1000 replicates. The NJ phylogenetic tree showed that all the PLD genes from the four higher plants divided into 6 well-supported clades (bootstrap values from 64% to 100%). Among these, the previously classified β and γ isoforms clustered closely together and were not explicitly separated from each other. Accordingly, the tree clades were classified into six subgroups, α, β/γ, δ, ε, ζ and φ (Figure 3). Among these, the α subgroup constituted the largest clade containing 19 members, and the β/γ subgroup formed the second largest clade containing 12 members (bootstrap value, 100%). Additionally, the β/γ and δ subgroups further clustered forming a larger clade and implying that they originated from a common ancestor by frequent gene duplication. Among these subgroups, the α, β/γ, δ, ε comprised C2-PLDs while the ζ and φ subgroups comprised PXPH-PLDs and SP-PLDs. Interestingly, although phylogenetically members of the ε subgroup comprised C2-PLDs, they appeared somewhat divergent from this class of PLDs and were indeed intermediary between the C2-PLDs and PXPH-PLDs. Distinct from the other C2-PLDs, PLDε appeared to possess the C2 structural fold, but this contained none of the acidic amino acid residues thought to be involved in Ca^2+ ^binding, suggesting that the phospholipid binding of PLDε is less Ca^2+^-dependent than the other C2-PLDs [19]. This feature of the PLDε C2 domain appeared to be conserved between Poplar, Grape and Arabidopsis. Surprisingly, PLDε does not appear to exist in rice.

Structural analyses can provide valuable information concerning duplication events when interpreting phylogenetic relationships within gene families. Thus, the exon/intron structure of each member of the PLD family was analyzed (right panel in Figure 3). The number of exons determined for members of the PLD gene family ranged from 2 in OsPLDα6 to 22 in PtPLD16. Most members within the individual subgroups shared similar intron/exon numbers and predicted coding sequence (CDS) lengths, consistent with the phylogenetic classification of the PLDs into the subgroups depicted in the left panel of Figure 3. For example, both β/γ and δ subgroups included members with 9-12 exons with CDS lengths of between 792 to 1296 codons, consistent with the observation that they originated by continuous gene duplication. Interestingly, the genes VvPLD9 and VvPLD10 appeared longer than the other members of the β/γ and δ subgroups because of the presence of a single long intron that contained repeated retrotransposon elements [20]. Similarly, members of the α and ε subgroups possessed 3-4 exons, with some introns extended by retrotransposon elements, suggesting that they also had a common ancestor. Members of the ζ subgroup which comprised PXPH-PLDs, were distinct from the C2-PLDs clade in that they possessed between 19 to 21 exons (except AtPLDζ2 that had 16 exons), suggesting an independent evolutionary lineage. Similarly, all members of the PXPH-PLD φ subgroup had 7 exons, also implying they originated via an evolutionary path separate to that of the C2-PLDs. Thus, the phylogenetic and gene structure analysis suggested that the 6 PLD subgroups originated from 4 ancestors *via *a series of gene duplications.

PLD genes from each of the subgroups were found in all four species of the higher plants examined with the exception of members of the two small subgroups, ε and φ, which were absent in the rice and Arabidopsis genomes, respectively. Presumably, the main subgroups of the plant PLD gene family were established before the dicot-monocot lineage parted and before further division of the dicotyledonous non-woody and woody herbaceous lineage. The majority of the PLD genes from Poplar (76.5%, 13/17) and Grape (90.9%, 10/11) clustered more closely together in the phylogenetic tree than they did with those from Arabidopsis and rice (Figure 3), suggesting that two woody plants had a closer evolutionary relationship than with the non-woody herbaceous dicot and the monocot [21]. Five pairs of Poplar PLD genes (PtPLD10 and PtPLD4, PtPLD17 and PtPLD15, PtPLD6 and PtPLD3, PtPLD13 and PtPLD2, and PtPLD16 and PtPLD8) formed 5 well-supported subclusters (bootstrap values of 100%) (left panel in Figure 3), indicating that they were evolutionarily very closely related. Each pair of genes in each of the 5 subclusters had very similar structures (right panel in Figure 3), indicating that they originated from relatively recent gene duplications. Four of these five subclusters also clustered relatively closely with a similar Grape PLD gene. At least one pair of Grape PLD genes (VvPLD7 and VvPLD8) clustered sufficiently closely to suggest that they too arose from a recent duplication event. This Grape subcluster also clustered closely with a PLD gene from Poplar. Collectively, these results indicate that frequent gene duplications occurred following the divergence of the Poplar and Grape species and that in Poplar this resulted in a rapid expansion of the size of the PLD gene family.

### Evolutionary patterns of PLD gene family in Arabidopsis, rice, Poplar and Grape

Segmental duplication, tandem duplication and transposition events such as retroposition and replicative transposition are the main reasons for gene family expansion [22]. Two tandem PLD gene duplications have previously been identified in Arabidopsis (AtPLDγ2-AtPLDγ1-AtPLDγ3) and rice (OsPLDα3-OsPLDα4-OsPLDα5) [6,11]. Chromosomal location analyses of the PLD gene family in Polar and Grape showed that the majority of the genes appeared randomly scattered throughout the genome with the exception of one pair of Grape PLD genes (VvPLD7/VvPLD8) which were tightly co-located and thus most likely resulted from a tandem duplication (Figure 2). This suggests that tandem duplication is not a major contributory event leading to the expansion of the PLD gene family in higher plants. Thus, we hypothesized that, at least in Arabidopsis, rice, Poplar and Grape, segmental duplication and transposition events may have played a more leading role in the evolution of the PLD gene family.

To validate this hypothesis, we first selected 13 paralogous PLD gene pairs from the phylogenetic tree (Figure 3) and subsequently explored the degree to which the protein-coding genes flanking each paralogous pair were similar. There were 5 pairs of paralogous PLD genes identified in the phylogenetic tree for Poplar (Figure 3). The identities of the genes flanking both sides of all 5 pairs of the paralogous Poplar PLD genes were found to be highly conserved (Table 3), suggesting that all of the paralogous PLD genes in Poplar arose from segmental duplication events. Similarly, in rice the protein-coding genes flanking each of the three pairs of PLD paralogous genes identified (OsPLDβ1/OsPLDβ2, OsPLDα1/OsPLDα2, and OsPLDδ2/OsPLDδ3) were found to be conserved (Table 3). To better explore the mechanisms of the PLD gene family expansion in Grape, a phylogenetic analysis of only Grape PLD genes was used to identify paralogous gene pairs (see Additional file 1). Two additional gene pairs (VvPLD1/VvPLD10 and VvPLD9/VvPLD11) were thus identified and protein-coding gene identity was found to be highly conserved in the regions flanking the genes VvPLD1 and VvPLD10. Similarly, in Arabidopsis one PLD gene pair (AtPLDα1/AtPLDα2) with conserved protein-coding genes in their flanking regions was identified (Table 3).

**Table 3:** Duplicated PLD genes and the number of conserved protein-coding genes flanking them in Arabidopsis, rice, Poplar and Grape

Duplicated PLD gene 1	Duplicated PLD gene 2	Number of conserved flanking protein-coding genes	Mean ks	Date (million years ago)
PtPLD10	PtPLD4	7	0.2120	11.65
PtPLD17	PtPLD15	2	0.2059	11.31
PtPLD6	PtPLD3	12	0.2451	13.47
PtPLD13	PtPLD2	5	0.2505	13.76
PtPLD16	PtPLD8	6	0.2274	12.49
OsPLDβ1	OsPLDβ2	3	0.9023	69.41
OsPLDα1	OsPLDα2	3	0.9971	76.70
OsPLDδ2	OsPLDδ3	1	0.9190	70.69
VvPLD10	VvPLD1	3	1.1491	88.39
AtPLDα1	AtPLDα2	3	0.7527	25.09

Taken together, these findings indicate that the mechanisms underlying the gene duplications that have contributed to the expansion of the PLD gene family differ between the four higher plants examined. In Poplar, segmental duplication accounted for the majority of the gene duplications identified. In evolutionary terms, most of these Poplar PLD gene duplications appeared to have occurred relatively recently and may be associated with novel functional divergence and adaptation. However, in Arabidopsis, rice and Grape, both segmental duplication and transposition events appear to have contributed to the duplication of the PLD genes. It is worth noting that some 41.4% of the Grape genome is composed of repetitive/transposable elements [20]. Thus, it is prudent to propose that transposition events could have been an important factor governing the expansion of PLD gene family in this species.

To estimate the evolutionary dates of the segmental duplication events, Ks was used as the proxy for time and the conserved protein-coding genes flanking the PLD gene pairs were thus subjected to Ks calculation (Table 3). The protein-coding genes flanking the 5 pairs of duplicated genes in Poplar had very consistent mean Ks values (from 0.2059 to 0.2505), suggesting that the segmental duplication events in this species occurred within the last 11.31 to 13.76 million years. This time period is subsequent to the time at which the evolutionary lineage of Poplar and Arabidopsis divided, circa 100-120 million years ago (Ma), and is consistent with the time (13 Ma) when a recent large scale genome duplication event is thought to have occurred in Poplar [23]. The implication is that, relative to other species, the rapid expansion of the PLD gene family in Poplar resulted from higher order genome level processes.

The PLD gene segmental duplication in Grape was estimated to have occurred about 25.09 Ma (mean Ks = 0.7527), which is similar to when this was observed in Poplar. The observation that there are fewer PLD genes in Grape compared to Poplar may be due to the fact that Grape experienced two genome wide duplication (GWD) events during evolution compared to three in Poplar [24,25].

For rice, the segmental duplication event was estimated to have occurred between 69.41 to 76.70 Ma, which is subsequent to the time of divergence of the monocots and eudicots (170-235 Ma), but precedent to the time of the origin of the grasses (55-70 Ma) [26-28]. The earliest observed segmental duplication event occurred in the PLD genes of Arabidopsis around 88.39 Ma. It is interesting, therefore, that despite similar levels of GWD, Arabidopsis has comparably fewer PLD genes than Poplar. It is likely that this may due to the fact that the Arabidopsis genome has subsequently suffered a high level of gene loss [20,29].

### Functional divergence and driving forces for genetic divergence

Site-specific shift rates (Type-I functional divergence) reflect the difference in the evolutionary rate of change of specific amino acid sites in proteins following gene duplication [30,31]. In order to detect the Type-I functional divergence occurring in the PLDs, we determined the differences in the site-specific evolutionary rates of amino acid changes between the C2-PLD and PXPH-PLD clades (Figure 3) using the program DIVERGE. The results showed a significant evidence of type I functional divergence between the C2-PLDs and PXPH-PLDs (θ_I _= 0.64, P < 0.01, see Additional file 2). When the threshold values of posterior probability (Q_k_) were set to either 0.80 or 0.90, 75 and 40 amino acid sites, respectively, were determined to be associated with the functional divergence of the C2- and PXPH-PLDs (see Additional file 2).

Positive Darwinian selection has been reported to be associated with gene duplication and functional divergence. To explore whether positive selection drove evolution of the PLD gene family, the coding regions of thirteen PLD gene paralogs from Arabidopsis, rice, Poplar and Grape were subjected to sliding window analyses. The nonsynonymous (dN)/synonymous substitution (dS) ratio (ω = dN/dS) is generally used to identify positive selection. A dN/dS (also known as Ka/Ks) ratio >1, <1 and = 1 indicates positive, negative, or purifying selection, and neutral evolution, respectively [32]. We calculated the dN/dS ratios for all the paralogs depicted in the phylogenetic tree reported in Figure 3 with a sliding window of 300 bp and a moving step of 50 bp. The resulting pairwise comparison data showed that all the paralogous genes have dN/dS ratios of <1 except for the comparisons OsPLDα4 vs. OsPLDα5 and OsPLDβ1 vs. OsPLDβ2 (see Additional file 3), strongly suggesting that the PLD gene family had mainly experienced strong purifying selection pressure. Here, the action of such purifying selection on the duplicated Poplar PLD genes supports the observation that the rapid expansion of the PLD family in this species resulted from higher order genome level processes. The gene pair, OsPLDα4 and OsPLDα5, clustered closely together (bootstrap values of 100%) and exhibited very similar exon/intron structures (Figure 3), suggesting that they were derived from relatively recent gene duplication event. The gene pair OsPLDβ1 and OsPLDβ2 also appeared to be similarly derived. Pairwise comparisons between OsPLDα4 and OsPLDα5 and between OsPLDβ1 and OsPLDβ2 exhibited ω values >1 in some regions, especially in the N termini of the proteins (see Additional file 3), suggesting that a more recent episode of positive selection has occurred after the gene duplication event.

To further investigate the evolutionary selection pressures acting on the PLDs, a site-specific model was formulated using the Codeml program of PAML 4.0 [33] with sequences from the C2-, PXPH- and SP-PLD clades. Consistent with the pairwise comparison results, when using the robust codon-substitution model in PAML, purifying selection was also determined to have acted on the C2-PLDs (see Additional file 4). Such a selection pressure may indicate that strong functional constraints have a bearing on the evolution of the C2-PLDs, supporting the notion that this group of the PLDs have important and essential roles in the regulation of plant cellular processes. Conversely, the concept that purifying selection is the main evolutionary mode of amino acid change in the C2-PLDs, along with the fact that the majority (12/13) of duplicated PLD genes belong to this clade, implies that C2-PLD gene duplication is unlikely to be associated with the formation of PLDs of either novel or divergent function.

In contrast, positive selection was observed to have occurred during the evolution of the PXPH-PLDs and SP-PLDs. Weak (ω = 1.34) and strong (ω = 8.14) positive selections were determined to have acted on the PXPH-PLDs and SP-PLDs, respectively. Although not reaching significant levels (posterior probabilities >0.90), one (site 627) and 3 (sites 3, 18 and 23) positively selected amino acid sites were identified in the PXPH- and SP-PLDs, respectively (see Additional file 4).

**Figure 4:**
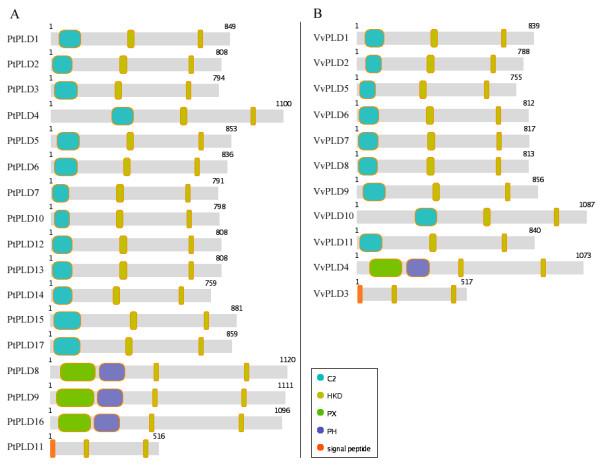
**Domain analysis and schematic diagram for domain structures of PLD genes in Poplar (A) and Grape (B)**. The C2 domain, PX domain, PH domain, HKD domain and Signal Peptide are represented by several rounded rectangles with different colours. The two HKD domains are represented by the rounded rectangle with same colour. The HKD domain near the N-terminal is named HKD1 and the other is named HKD2.

Plants possess relatively few PXPH- and SP-PLD genes in comparison to the numbers of C2-PLD genes found in their genomes (Figure 4). Thus, the positive selection that has acted on the PXPH- and SP-PLD genes may imply that their functional diversification has resulted from the need to adapt to a changing environment.

### Domains and Motifs analyses in PLD gene family

The proteins encoded by the newly identified Poplar and Grape PLD genes were subjected to protein domain analyses. The program hmmpfam in HMMer [34] was used initially to identify the major domains of the PLD proteins. Such domain analyses for Arabidopsis and rice PLDs has been previously performed [6,11]. Here, the analyses showed that the all the PLDs in Poplar and Grape possessed the two characteristic and structurally conserved HKD domains essential for their lipase activity. As in the case of Arabidopsis and rice, the Poplar and Grape PLDs could be classified into the three subgroups (C2-, PXPH- and SP-PLD), based on the presence of the subgroup-specific domains (Figure 4). As expected, in their N-terminal regions the C2-PLDs contained one C2 domain while the PXPH-PLDs contained both PX and PH domains and an N-terminal signal peptide was identified in each of the SP-PLDs (Figure 4).

**Figure 5:**
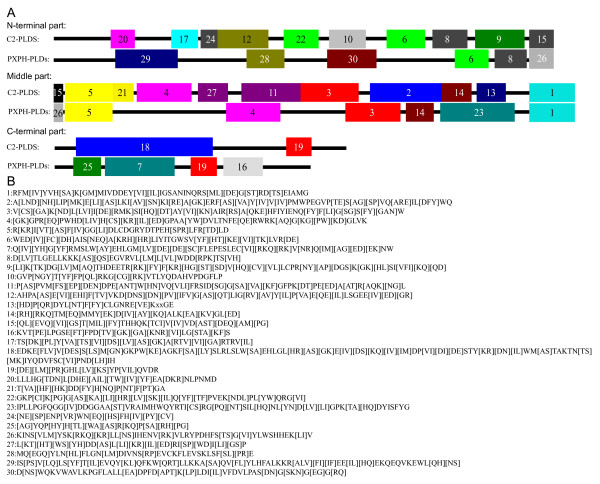
**MEME/MAST domain analysis and schematic diagram for main motif structures of PLD genes**. Panel A shows the motif structures of the PLD genes in three parts: the N-terminal region before the first HKD domain, the middle region including the region in and between the two HKD domains, the C-terminal region after the second HKD domain. Panel B shows the regular-expression sequences of the thirty motifs.

Such domain search tools are suitable for defining the presence or absence of roughly recognisable domains, but they are unable to recognize either smaller individual motifs or more divergent patterns. Thus, we used the motif search tool MEME/MAST to mine for more detailed motif information (see Additional file 5) in the PLDs of the four higher plants examined. The thirty motifs identified by MEME were annotated by InterProScan [35]. The result showed that, except for motifs 20, 25, 27 and 29, the majority of these domains were functionally associated with PLD activity (see Additional file 6). For analytical convenience, we divided the data into parts covering three regions of the PLDs: the N-terminal region before the first HKD domain, the middle region including the region in and between the two HKD domains, and the C-terminal region after the second HKD domain (Figure 5).

The N-terminal region of the C2-PLDs contained 10 motifs, compared with 6 motifs in the same region of the PXPH-PLDs (Figure 5A). Four motifs found in the N-termini of the C2-PLDs (20, 17, 24 and 12) appeared specific to this PLD clade (see Additional file 7) and have been suggested to take part in the formation of an eight βstrand switch involved in Ca^2+^-binding [36]. Motifs 28 and 29 appeared to be specific to the PX domain and motif 30 to the PH domain of the PXPH-PLDs (see Additional file 8), and are thought to be associated with the binding of phosphatidylinositol lipids [36]. One observed exception was AtPLDγ3 that contained an additional motif, 13, in the C2 domain. Additionally, in OsPLDα7, PtPLD10, PtPLD14, VvPLD5, AtPLDα3, AtPLDε and AtPLDγ2 the C2 domain appeared to have lost either one or two of the four motifs mentioned above that are associated with the binding of Ca^2+ ^(see Additional file 5). The N-terminal region of the C2-PLDs, the region behind C2 domain (referred to as the post-C2 region) usually included the 6 motifs 22, 10, 6, 8, 9, and 15. A degree of loss of some of these motifs was observed in some of the C2-PLDs from each of the four species examined. For example, AtPLDε, VvPLD5, VvPLD2, PtPLD14, PtPLD17, PtPLD6, PtPLD3 and OsPLDα7 appear to have lost either one or both of motifs 22 and 15 (see Additional file 5). In contrast, no motif loss was observed in the PXPH-PLDs, possibly due to the small number of members of this subgroup and the relatively small number of motifs in the N-terminal region of these PLDs. Both the C2-PLDs and PXPH-PLDs shared two conserved motifs, 6 and 8, implying a crucial role for these in PLD function.

**Figure 6:**
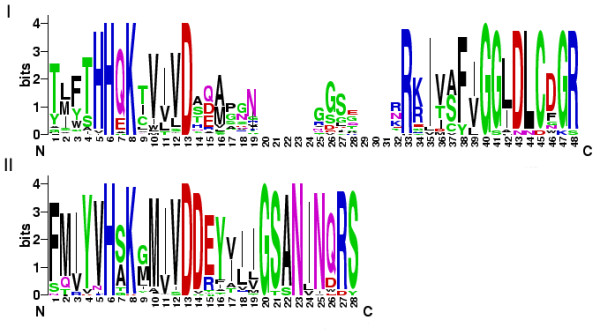
**Sequence logos for the two HKD domains (I: HKD1, II: HKD2)**. Numbers on the x-axis represent the sequence positions in respect HKD domain. The y-axis represents the information content measured in bits.

The middle region of the PLDs contained 11 and 7 motifs in the C2-PLDs and PXPH-PLDs, respectively. There appeared to be a relatively higher level of conservation in this region between the C2-PLDs and PXPH-PLDs as they shared 5 motifs (5, 4, 3, 14 and 1). The middle region of the C2-PLDs and PXPH-PLDs started from the C-terminal ends of motifs 15 and 26, respectively, and ended with motif 1 that formed the HKD2 domain. The C-terminal sequences of motifs 15 and 26 are identical and, together with motif 5, formed the HKD1 domain (see Additional file 9). Thus, these two sections of the middle region appeared identical in both the C2-PLDs and PXPH-PLDs (Figure 5A). Comparative sequence alignment of the two HKD domains revealed that the HKD1 domain sequence was relatively more diverse than that of the HKD2 domain (Figure 6, see Additional file 9). Both the HKD1 and HKD2 domains contained three highly conserved amino acids (6H, 8K and 13D), implying that these have a key functional importance within these domains. It would appear that the two HKD domains have co-evolved within the PLD family. Phylogenetic analysis of the HKD1 and HKD2 domains, respectively, produced two trees that exhibited similar topology (see Additional file 10) to that revealed by the same analysis of the PLD gene family. The HKD domain trees clustered similarly into 5 subgroups, with gene members clustering in an almost identical fashion to that observed when the full length PLDs were so analysed.

Three other motifs (4, 3 and 14) in the middle region were also shared by both the C2-PLDs and PXPH-PLDs. Motif 4 contained a regular-expression sequence "[GK]GPR[EQ]PWHD[LIV]H[CS][KR][IL][ED]GPA[YW]DVLTNFE[QE]RWRK[AQ]G[G][PW][KD]GLVK" (Figure 5B) which is thought to form the binding site of PIP_2_. Variations in the sequence of this motif exhibit different PIP_2 _binding affinity [8,37,38]. Sequence alignment of motif 4 from the individual PLDs showed that 73.2% (30/41) of the amino acid sites were highly conserved with 10 of them being fully conserved, suggesting that they may play an essential role in the binding of both C2-PLDs and PXPH-PLDs to PIP_2 _(see Additional file 11). Motif 3 contained a regular-expression sequence "IYIENQ[FY]F" (Figure 5B). The seventh amino acid of this regular-expression sequence, Phe (F), appeared in all PXPH-PLDs, but was often substituted by Tyr (Y) in the C2-PLDs (see Additional file 12). This short sequence was only found in the PLD family members, and has been postulated to increase the rate of catalysis and ensure substrate specificity [8]. This suggests that the sequence "IYIENQ[FY]F" may be almost as critical as the HKD motif for PLD activity [39,40]. In addition, motif 14 was also found in both the C2-PLDs and PXPH-PLDs (see Additional file 13). Four amino acid sites in this motif were shown to be highly conserved, especially the eighth amino acid, tyrosine, which was fully conserved. With the exception of these four conserved amino acid sites, the remainder of motif 14 exhibited a high degree of sequence polymorphism.

Apart from these shared motifs, the C2-PLDs and PXPH-PLDs possessed a number of clade-specific motifs within their middle regions. The C2-PLDs had 5 such motifs (21, 27, 11, 2 and13) and the PXPH-PLDs one motif, 23 (Figure 5A). The C2-PLD-specific motif 2 contained a core triplet of amino acids, "ERF", followed by a highly conserved hydrophobic region, "VYVVV" (see Additional file 14). in AtPLDα1, this motif was reported as being able to bind to the Gα subunit of the Arabidopsis heterotrimeric G protein [41]. When the sequences of motif 2 from the different C2-PLDs were aligned, the ERF triplet appeared to be relatively more conserved than the "VYVVV" region. However, mutations that have occurred in OsPLDα7, AtPLDγ2, and OsPLDδ2 changed the second residue of the ERF triplet from the basic amino acid R into the non-charged amino acids S, N and Q, respectively, implying a possible change in the ability of these PLDs to bind to the heterotrimeric G protein Gα subunit [41].

In the C-terminal region, the C2-PLDs contained 4 motifs (25, 7, 19 and 16) and the PXPH-PLDs 2 motifs (18 and 19) (Figure5), thus sharing the single motif 19.

Overall, 8 motifs (6, 8, 5, 4, 3, 14, 1 and 19) were shown to be shared by both the C2- and PXPH-PLDs subgroups, implying that they are likely to be necessary for PLD function. The majority of these conserved motifs appeared to exist in the middle region of the PLDs, strongly suggesting that the two HKD domains in this region play a key role in the lipase activity of the PLDs.

## Conclusion

In this study, we have provided a genome-wide identification and analysis of the PLD gene family in Poplar and Grape. Eighteen and 11 members of the PLD gene family were identified in Poplar and Grape, respectively. Phylogenetic and gene structure analyses showed that the PLD gene family can be divided into 6 subgroups (α, β/γ, δ, ε, ζ, and φ) and that these 6 PLD subgroups originated from 4 original ancestors through a series of gene duplications. Phylogenetically, the majority of the PLD genes from Poplar (82.8%, 14/17) and Grape (90.9%, 10/11) clustered particularly closely, suggesting a close evolutionary relationship between these two species. Five pairs of duplicated PLD genes were identified in Poplar, more than those identified in Grape, suggesting that frequent gene duplication occurred after the species diverged resulting in a rapid expansion of the PLD gene family in Poplar. The majority of gene duplications in Poplar appeared to have been caused by segmental duplication, distinguishing it from the other three plant species, Arabidopsis, rice and Grape, where both segmental duplication and transposition events appeared to have contributed to the duplication of the PLD genes. Furthermore, the PLD gene duplications in Poplar were estimated to have occurred between 11.31 to 13.76 Ma, substantially later than the time when duplications occurred in the other three plant species (25.09 to 88.39 Ma). Adaptive evolution analysis showed that purifying selection has driven evolution of the C2-PLDs, whereas positive selection has contributed, at least in part, to the evolution of the remaining PLDs, especially after gene duplication.

The PLD gene family is divided into two main subfamilies, C2-PLDs and PXPH-PLDs, and one smaller subfamily, SP-PLDs. Motif analyses show that the C2-PLDs and PXPH-PLDs generally contain 23 and 17 motifs, respectively. Among these, 8 motifs were shared by both the C2- and PXPH-PLDs subfamilies, implying that they may be necessary for PLD function. The majority of these shared motifs exist in the middle region of the PLDs, suggesting that the two HKD domains also play a core role in PLD activity.

This detailed analysis of the PLD gene family in these two woody plants has provided the data that will form the basis for future hypothesis-driven experiments involving either loss- or gain-of-function studies aimed at clarifying the role of the different PLDs in the growth, development and survival of Poplar and Grape. Thus, this new knowledge of the PLD gene family in these species may lead to the possibility of modulating PLD gene expression and function in order to control specific aspects of the physiology and development of woody plants.

## Methods

### Identification of PLD gene families in Poplar and Grape

To identify members of the PLD gene family in Poplar and Grape, multiple database searches were performed. Arabidopsis PLD gene sequences were retrieved from http://www.arabidopsis.org and used as queries to perform repetitive blast searches against the Poplar Genome (V1.1) database http://genome.jgi-psf.org/Poptr1_1/Poptr1_1.home.html and the Genoscope Genome Project Grape genome database http://www.cns.fr/. Blast searches were also performed against nucleic acid sequence data repositories at the National Center for Biotechnology Information (NCBI, http://www.ncbi.nlm.nih.gov. Genes annotated as "Phospholipases D" or "PLD" were also collected by keyword searches in Genbank. Additionally, a Hidden Markov Model (HMM) search was performed in the proteome databases of Poplar and Grape using HKD domain HMM profiles (PFAM, PF00614). Profile searches were performed using the HMMER 2.3.2 software package [34]. All protein sequences derived from the candidate PLD genes collected were examined using the domain analysis programs, PFAM http://pfam.sanger.ac.uk/ and SMART http://smart.embl-heidelberg.de/ with the default cut off parameters. Gene sequences with two HKD domains were considered to be members of the PLD gene family. Pseudogenes were determined according to their gene annotation or when their coding sequences were obviously terminated by premature stop codons.

### Sequence and phylogenetic analyses of PLD gene family

PLD gene sequences were aligned using the program Clustal X with BLOSUM30 as the protein weight matrix. The program MUSCLE (version 3.52) was also used to perform multiple sequence alignments to confirm the Clustal X data output [42]. Phylogenetic trees based on the protein sequences of the PLDs were constructed using the neighbor-joining (NJ) method of the program MEGA4 [43] with p-distance and the complete deletion option parameters engaged. The reliability of the trees obtained was tested using bootstrapping with 1000 replicates. Images of the phylogenetic trees were also drawn using MEGA4.

### Chromosomal location and Gene structure of PLD genes

PLD gene chromosomal locations were determined using the Poplar genome browser http://genome.jgi-psf.org/Poptr1_1/optr1_1.home.html and Grape genome browser http://www.cns.fr/externe/Genome Browser/Vitis/, respectively. Gene intron/extron structure information was collected from the genome annotations of Poplar and Grape from NCBI.

### Protein Motif analysis

In order to investigate protein motifs in more detail, the PLD protein sequences were analyzed using the MEME/MAST software http://meme.sdsc.edu/[44,45]. The functional annotation of the identified motifs was implemented by InterProScan http://www.ebi.ac.uk/Tools/InterProScan/.

### Analysis of PLD gene expansion patterns

Segmental (chromosomal segments) duplication, tandem duplication (duplications in a tandem pattern) and transposition events result in gene family expansion [46]. Transposition occurs when a segment from one chromosome becomes unaligned with the corresponding segment from the other chromosome. Because it is difficult to identify transposition events based on gene sequence analysis, in this study we focused on the processes of segmental and tandem duplication. To categorize expansion of the PLD gene family, we examined the chromosomal locations of all members of this family in Arabidopsis, rice, Poplar and Grape. Tandem duplication was characterized by multiple gene family members occurring within either the same or neighboring intergenic regions. A method similar to that of Maher *et al*. [47] was used to identify segmental duplications. First paralogous PLD genes were identified at the terminal nodes of the phylogenetic tree. Next, 10 protein-coding genes upstream and downstream of each pair of paralogs were obtained from the annotated genomes of Arabidopsis, rice, Poplar and Grape. Lastly, the similarity between the genes flanking one PLD gene and those flanking the other PLD gene in each pair of paralogs was determined. A pair of paralogous PLD genes was considered to have originated from a duplication event if both resided within a region of conserved protein-coding genes.

### Calculating Ks to date the duplication events and adaptive evolution analysis of PLD gene family

Pairwise alignment of nucleotide sequences of PLD paralogs was performed using Clustal X1.83. Gaps in the alignments were removed manually by Bioedit. The Ka and Ks values of the paralogous genes were estimated by the program K-Estimator 6.0 [48]. To better explain the patterns of macro-evolution, estimates of the evolutionary rates were considered extremely useful. Assuming a molecular clock, the synonymous substitution rates (Ks) of duplicated genes would be expected to be similar over time [49]. Thus, Ks could be used as the proxy for time and the conserved flanking protein-coding genes was used to estimate the dates of the segmental duplication events. The mean Ks value was calculated for each of duplicated gene pairs and then used to date the duplication events. Ks values greater than 2.0 were discarded in order to avoid the risk of saturation. The Ks values were then used to calculate the approximate date of the duplication event(T = Ks/2λ), assuming clock-like rates (λ) of synonymous substitution of 1.5 × 10 ^-8 ^substitutions/synonymous site/year for Arabidopsis [50], 6.5 × 10 ^-9 ^for rice [51], 9.1 × 10 ^-9 ^for Poplar [52], and 6.5 × 10 ^-9 ^for Grape [53]. To investigate whether Darwinian positive selection was involved in driving gene divergence after duplication, first Sliding Window analysis (300 bp window, 50 bp slide) was performed on the coding regions of paralogous PLD genes from the four plant species studied and was then used to calculate the Ka/Ks ratio. Subsequently, the codon-based site model of codeml in PAML [33] was used to perform adaptive evolution analysis on the three different types of PLD genes separately.

## Authors' contributions

QL carried out the computational analyses and wrote in-house program. QL and CZ interpreted the results and wrote the manuscript. XH was involved in planning of experiments and headed the project. XH and YY revised the final version of the manuscript. All authors read and approved the final manuscript.

## Supplementary Material

Additional file 1 **Phylogenetic tree of Grape PLD genes. ** The gene pairs covered with shaded boxes represent the paralogous genes in the Grape phylogenetic tree. Click here for file

Additional file 2 **Functional divergence estimated from pairwise comparison between C2-PLDs and PXPH-PLDs**. Click here for file

Additional file 3 **The ka/ks ratios for PLD paralogous genes in Arabidopsis, rice, Poplar and Grape**. Click here for file

Additional file 4 **Parameter estimations and likelihood ratio tests for the site models in codeml**. Click here for file

Additional file 5 **Thirty putative motifs identified in all PLD gene family members in the four higher plants by MEME/MAST software**. Different motifs are indicated by different colors. Names of all the members from different subfamilies and combined P values are shown on the left side of the figure and motif sizes are indicated at the bottom of the figure. Click here for file

Additional file 6 **Function annotations of the motifs.** Missing database hits of function annotations in motif 20, 25, 27 and 29 by InterProScan. Click here for file

Additional file 7 **Alignment of sequences of C2 domain of PLD genes in Arabidopsis, rice, Poplar and Grape**. Black and gray shading indicate identical and conserved amino acid residues present in more than 50% of the aligned sequences, respectively. The colour bars and numbers above the sequence alignment represent MEME motifs. Click here for file

Additional file 8 **Alignment of sequences of PX domain (A) and PH domain (B) of PLD genes in Arabidopsis, rice, Poplar and Grape.**  Black and gray shading indicate identical and conserved amino acid residues present in more than 50% of the aligned sequences, respectively. The colour bar and numbers above the sequence alignment represent MEME motifs. Click here for file

Additional file 9 **Alignment of sequences of HKD1 (A) and HKD2 (B) of PLD genes in Arabidopsis, rice, Poplar and Grape**. Black and gray shading indicate identical and conserved amino acid residues present in more than 50% of the aligned sequences, respectively. The colour bar and numbers above the sequence alignment represent MEME motifs. Click here for file

Additional file 10 **Phylogenetic trees of the HKD1 domain(A) and HKD2 domain(B) sequences, respectively**. The clades marked with the same color in the two trees represent the same kind of subgroup. Click here for file

Additional file 11 **Alignment of sequences of MEME motif 4 in PLD genes in Arabidopsis, rice, Poplar and Grape.**  Black and gray shadings indicate identical and conserved amino acid residues present in more than 50% of the aligned sequences, respectively. The colour bar and number above the sequence alignment represent MEME motifs. Click here for file

Additional file 12 **Alignment of sequences of MEME motif 3 in PLD genes in Arabidopsis, rice, Poplar and Grape**. Black and gray shadings indicate identical and conserved amino acid residues present in more than 50% of the aligned sequences, respectively. The colour bar and number above the sequence alignment represent MEME motifs. The sites marked by red boxes represent the "IYIENQ[FY]F" motif. Click here for file

Additional file 13 **Alignment of sequences of MEME motif 14 in PLD genes in Arabidopsis, rice, Poplar and Grape**. Black and gray shadings indicate identical and conserved amino acid residues present in more than 50% of the aligned sequences, respectively. The colour bar and number above the sequence alignment represent MEME motifs. Click here for file

Additional file 14 **Alignment of sequences of MEME motif 2 in PLD genes in Arabidopsis, rice, Poplar and Grape**. Black and gray shadings indicate identical and conserved amino acid residues present in more than 50% of the aligned sequences, respectively. The colour bar and number above the sequence alignment represent MEME motifs. The sites marked by red boxes represent the DRY motif. Click here for file

## References

[B1] Wang X Regulatory functions of phospholipase D and phosphatidic acid in plant growth, development, and stress responses Plant Physiol 2005 139 2 566 573 10.1104/pp.105.068809 16219918 PMC1255977

[B2] Munnik T Phosphatidic acid: an emerging plant lipid second messenger Trends Plant Sci 2001 6 5 227 233 10.1016/S1360-1385(01)01918-5 11335176

[B3] DJ H IL C A new phospholipidsplitting enzyme specific for the ester linkage between the nitrogenous base and the phosphoric acid grouping J Biol Chem 1947 169 669 705 20259103

[B4] Cockcroft S Ca2+-dependent conversion of phosphatidylinositol to phosphatidate in neutrophils stimulated with fMet-Leu-Phe or ionophore A23187 Biochim Biophys Acta 1984 795 1 37 46 6432054 10.1016/0005-2760(84)90102-4

[B5] Bocckino SB Blackmore PF Wilson PB Exton JH Phosphatidate accumulation in hormone-treated hepatocytes via a phospholipase D mechanism J Biol Chem 1987 262 31 15309 15315 3117799

[B6] C Q X W The Arabidopsis phospholipase D family: characterization of a Ca2+-independent and phosphatidylcholine-selective PLDζ1 with distinct regulatory domains Plant Physiol 2002 128 1057 1068 10.1104/pp.010928 11891260 PMC152217

[B7] Qin W Pappan K Wang X Molecular heterogeneity of phospholipase D (PLD). Cloning of PLDgamma and regulation of plant PLDgamma, -beta, and -alpha by polyphosphoinositides and calcium J Biol Chem 1997 272 45 28267 28273 10.1074/jbc.272.45.28267 9353280

[B8] McDermott M Wakelam MJ Morris AJ Phospholipase D Biochem Cell Biol 2004 82 1 225 253 10.1139/o03-079 15052340

[B9] Sharma S Gupta MN Purification of phospholipase D from Dacus carota by three-phase partitioning and its characterization Protein Expr Purif 2001 21 2 310 316 10.1006/prep.2000.1357 11237693

[B10] Qin C Wang C Wang X Kinetic analysis of Arabidopsis phospholipase Ddelta. Substrate preference and mechanism of activation by Ca2+ and phosphatidylinositol 4,5-biphosphate J Biol Chem 2002 277 51 49685 49690 10.1074/jbc.M209598200 12397060

[B11] Li G Lin F Xue HW Genome-wide analysis of the phospholipase D family in Oryza sativa and functional characterization of PLD beta 1 in seed germination Cell Res 2007 17 10 881 894 10.1038/cr.2007.77 17876344

[B12] Yamaguchi T Kuroda M Yamakawa H Ashizawa T Hirayae K Kurimoto L Shinya T Shibuya N Suppression of a phospholipase D gene, OsPLDbeta1, activates defense responses and increases disease resistance in rice Plant Physiol 2009 150 1 308 319 10.1104/pp.108.131979 19286937 PMC2675732

[B13] Testerink C Munnik T Phosphatidic acid: a multifunctional stress signaling lipid in plants Trends Plant Sci 2005 10 8 368 375 10.1016/j.tplants.2005.06.002 16023886

[B14] Koonin EV A duplicated catalytic motif in a new superfamily of phosphohydrolases and phospholipid synthases that includes poxvirus envelope proteins Trends Biochem Sci 1996 21 7 242 243 8755242

[B15] JH E Phospholipase D-structure, regulation and function Rev Physiol Biochem Pharmacol 2002 1 1 94 10.1007/BFb0116585 11987824

[B16] Kopka J Pical C Hetherington AM Muller-Rober B Ca2+/phospholipid-binding (C2) domain in multiple plant proteins: novel components of the calcium-sensing apparatus Plant Mol Biol 1998 36 5 627 637 10.1023/A:1005915020760 9526495

[B17] van Leeuwen W Okresz L Bogre L Munnik T Learning the lipid language of plant signalling Trends Plant Sci 2004 9 8 378 384 10.1016/j.tplants.2004.06.008 15358268

[B18] Elias M Potocky M Cvrckova F Zarsky V Molecular diversity of phospholipase D in angiosperms BMC Genomics 2002 3 1 2 10.1186/1471-2164-3-2 11876823 PMC77410

[B19] Hong Y Devaiah SP Bahn SC Thamasandra BN Li M Welti R Wang X Phospholipase D epsilon and phosphatidic acid enhance Arabidopsis nitrogen signaling and growth Plant J 2009 58 3 376 387 10.1111/j.1365-313X.2009.03788.x 19143999 PMC4076113

[B20] Jaillon O Aury JM Noel B Policriti A Clepet C Casagrande A Choisne N Aubourg S Vitulo N Jubin C The grapevine genome sequence suggests ancestral hexaploidization in major angiosperm phyla Nature 2007 449 7161 463 467 10.1038/nature06148 17721507

[B21] Hedges SB The origin and evolution of model organisms Nat Rev Genet 2002 3 11 838 849 10.1038/nrg929 12415314

[B22] Kong H Landherr LL Frohlich MW Leebens-Mack J Ma H dePamphilis CW Patterns of gene duplication in the plant SKP1 gene family in angiosperms: evidence for multiple mechanisms of rapid gene birth Plant J 2007 50 5 873 885 10.1111/j.1365-313X.2007.03097.x 17470057

[B23] Sterck L Rombauts S Jansson S Sterky F Rouze P Van de Peer Y EST data suggest that poplar is an ancient polyploid New Phytol 2005 167 1 165 170 10.1111/j.1469-8137.2005.01378.x 15948839

[B24] Tuskan GA Difazio S Jansson S Bohlmann J Grigoriev I Hellsten U Putnam N Ralph S Rombauts S Salamov A The genome of black cottonwood, Populus trichocarpa (Torr. & Gray) Science 2006 313 5793 1596 1604 10.1126/science.1128691 16973872

[B25] Bowers JE Chapman BA Rong J Paterson AH Unravelling angiosperm genome evolution by phylogenetic analysis of chromosomal duplication events Nature 2003 422 6930 433 438 10.1038/nature01521 12660784

[B26] Wolfe KH Gouy M Yang YW Sharp PM Li WH Date of the monocot-dicot divergence estimated from chloroplast DNA sequence data Proc Natl Acad Sci USA 1989 86 16 6201 6205 10.1073/pnas.86.16.6201 2762323 PMC297805

[B27] Crane PR Friis EM Pedersen KR The origin and early diversification of angiosperms Nature 1995 374 6517 27 33 10.1038/374027a0

[B28] Kellogg EA Evolutionary history of the grasses Plant Physiol 2001 125 3 1198 1205 10.1104/pp.125.3.1198 11244101 PMC1539375

[B29] Ku HM Vision T Liu J Tanksley SD Comparing sequenced segments of the tomato and Arabidopsis genomes: large-scale duplication followed by selective gene loss creates a network of synteny Proc Natl Acad Sci USA 2000 97 16 9121 9126 10.1073/pnas.160271297 10908680 PMC16832

[B30] Gu X Statistical methods for testing functional divergence after gene duplication Mol Biol Evol 1999 16 12 1664 1674 10605109 10.1093/oxfordjournals.molbev.a026080

[B31] Gu X Maximum-likelihood approach for gene family evolution under functional divergence Mol Biol Evol 2001 18 4 453 464 11264396 10.1093/oxfordjournals.molbev.a003824

[B32] Li WH Gojobori T Rapid evolution of goat and sheep globin genes following gene duplication Mol Biol Evol 1983 1 1 94 108 6599963 10.1093/oxfordjournals.molbev.a040306

[B33] Yang Z PAML 4: phylogenetic analysis by maximum likelihood Mol Biol Evol 2007 24 8 1586 1591 10.1093/molbev/msm088 17483113

[B34] Eddy SR Profile hidden Markov models Bioinformatics 1998 14 9 755 763 10.1093/bioinformatics/14.9.755 9918945

[B35] Hunter S Apweiler R Attwood TK Bairoch A Bateman A Binns D Bork P Das U Daugherty L Duquenne L InterPro: the integrative protein signature database Nucleic Acids Res 2009 37 Database D211 215 10.1093/nar/gkn785 18940856 PMC2686546

[B36] Sutton RB Davletov BA Berghuis AM Sudhof TC Sprang SR Structure of the first C2 domain of synaptotagmin I: a novel Ca2+/phospholipid-binding fold Cell 1995 80 6 929 938 10.1016/0092-8674(95)90296-1 7697723

[B37] Pappan K Qin W Dyer JH Zheng L Wang X Molecular cloning and functional analysis of polyphosphoinositide-dependent phospholipase D, PLDbeta, from Arabidopsis J Biol Chem 1997 272 11 7055 7061 10.1074/jbc.272.11.7055 9054397

[B38] Pappan K Austin-Brown S Chapman KD Wang X Substrate selectivities and lipid modulation of plant phospholipase D alpha, -beta, and -gamma Arch Biochem Biophys 1998 353 1 131 140 10.1006/abbi.1998.0640 9578608

[B39] Sung TC Roper RL Zhang Y Rudge SA Temel R Hammond SM Morris AJ Moss B Engebrecht J Frohman MA Mutagenesis of phospholipase D defines a superfamily including a trans-Golgi viral protein required for poxvirus pathogenicity EMBO J 1997 16 15 4519 4530 10.1093/emboj/16.15.4519 9303296 PMC1170078

[B40] Wang C Wang X A novel phospholipase D of Arabidopsis that is activated by oleic acid and associated with the plasma membrane Plant Physiol 2001 127 3 1102 1112 10.1104/pp.010444 11706190 PMC129279

[B41] J Z X W Arabidopsis phospholipase Da1 interacts with the heterotrimeric G-protein a subunit through a motif analogous to the DRY motif in G-protein-coupled receptors J Biol Chem 2004 279 1794 1800 14594812 10.1074/jbc.M309529200

[B42] Edgar RC MUSCLE: multiple sequence alignment with high accuracy and high throughput Nucleic Acids Res 2004 32 5 1792 1797 10.1093/nar/gkh340 15034147 PMC390337

[B43] Kumar S Nei M Dudley J Tamura K MEGA: a biologist-centric software for evolutionary analysis of DNA and protein sequences Brief Bioinform 2008 9 4 299 306 10.1093/bib/bbn017 18417537 PMC2562624

[B44] Bailey TL Elkan C Fitting a mixture model by expectation maximization to discover motifs in biopolymers Proc Int Conf Intell Syst Mol Biol 1994 2 28 36 7584402

[B45] Bailey TL Gribskov M Combining evidence using p-values: application to sequence homology searches Bioinformatics 1998 14 1 48 54 10.1093/bioinformatics/14.1.48 9520501

[B46] Cannon SB Mitra A Baumgarten A Young ND May G The roles of segmental and tandem gene duplication in the evolution of large gene families in Arabidopsis thaliana BMC Plant Biol 2004 4 10 10.1186/1471-2229-4-10 15171794 PMC446195

[B47] Maher C Stein L Ware D Evolution of Arabidopsis microRNA families through duplication events Genome Res 2006 16 4 510 519 10.1101/gr.4680506 16520461 PMC1457037

[B48] Comeron JM K-Estimator: calculation of the number of nucleotide substitutions per site and the confidence intervals Bioinformatics 1999 15 9 763 764 10.1093/bioinformatics/15.9.763 10498777

[B49] Shiu SH Karlowski WM Pan R Tzeng YH Mayer KF Li WH Comparative analysis of the receptor-like kinase family in Arabidopsis and rice Plant Cell 2004 16 5 1220 1234 10.1105/tpc.020834 15105442 PMC423211

[B50] Blanc G Wolfe KH Widespread paleopolyploidy in model plant species inferred from age distributions of duplicate genes Plant Cell 2004 16 7 1667 1678 10.1105/tpc.021345 15208399 PMC514152

[B51] Yu J Wang J Lin W Li S Li H Zhou J Ni P Dong W Hu S Zeng C The Genomes of Oryza sativa: a history of duplications PLoS Biol 2005 3 2 e38 10.1371/journal.pbio.0030038 15685292 PMC546038

[B52] Lynch M Conery JS The evolutionary fate and consequences of duplicate genes Science 2000 290 5494 1151 1155 10.1126/science.290.5494.1151 11073452

[B53] Gaut BS Morton BR McCaig BC Clegg MT Substitution rate comparisons between grasses and palms: synonymous rate differences at the nuclear gene Adh parallel rate differences at the plastid gene rbcL Proc Natl Acad Sci USA 1996 93 19 10274 10279 10.1073/pnas.93.19.10274 8816790 PMC38374

