# Supplementary figures and images for: Genome-wide and molecular evolution analyses of the phospholipase D gene family in Poplar and Grape
Source: BMC Plant Biol. 2010 Jun 18;10:117. doi: 10.1186/1471-2229-10-117 (PMC3095279; doi:10.1186/1471-2229-10-117)

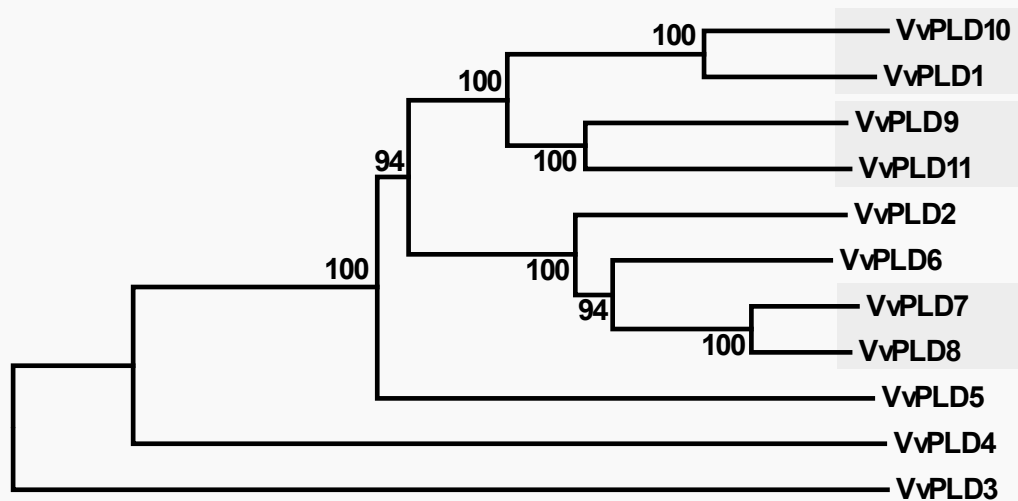

Supplement: Additional file 1 — Phylogenetic tree of Grape PLD genes. The gene pairs covered with shaded boxes represent the paralogous genes in the Grape phylogenetic tree. [file 1471-2229-10-117-S1.PDF]

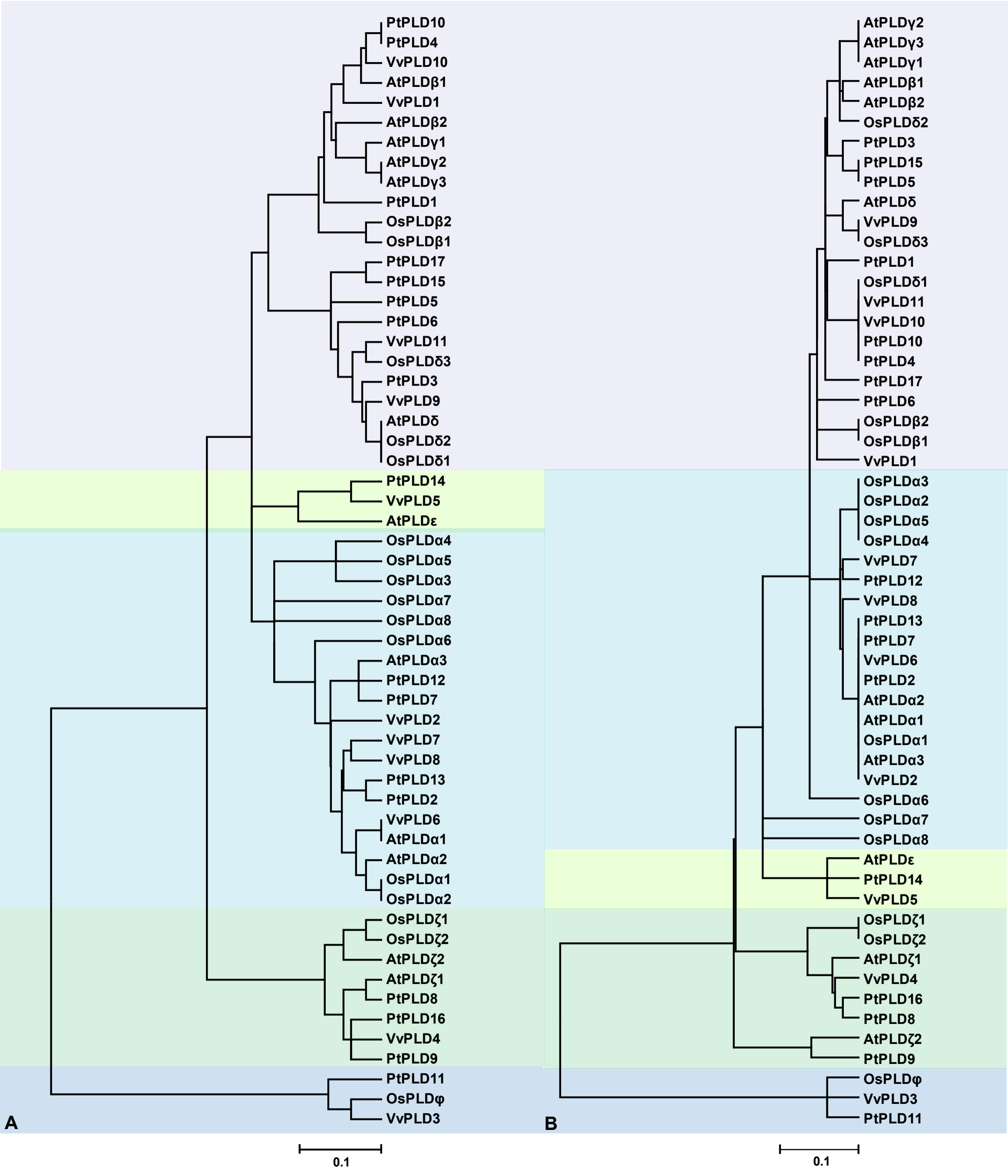

Supplement: Additional file 2 — Functional divergence estimated from pairwise comparison between C2-PLDs and PXPH-PLDs. [file 1471-2229-10-117-S2.PDF]

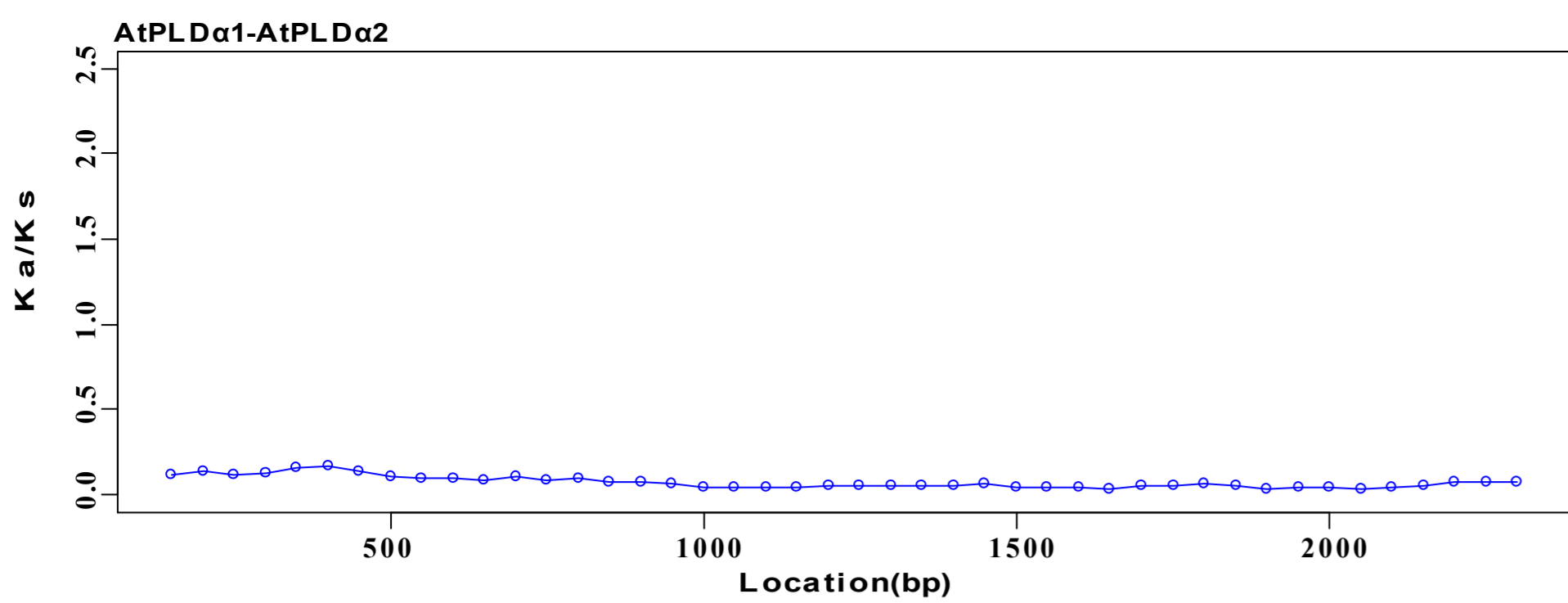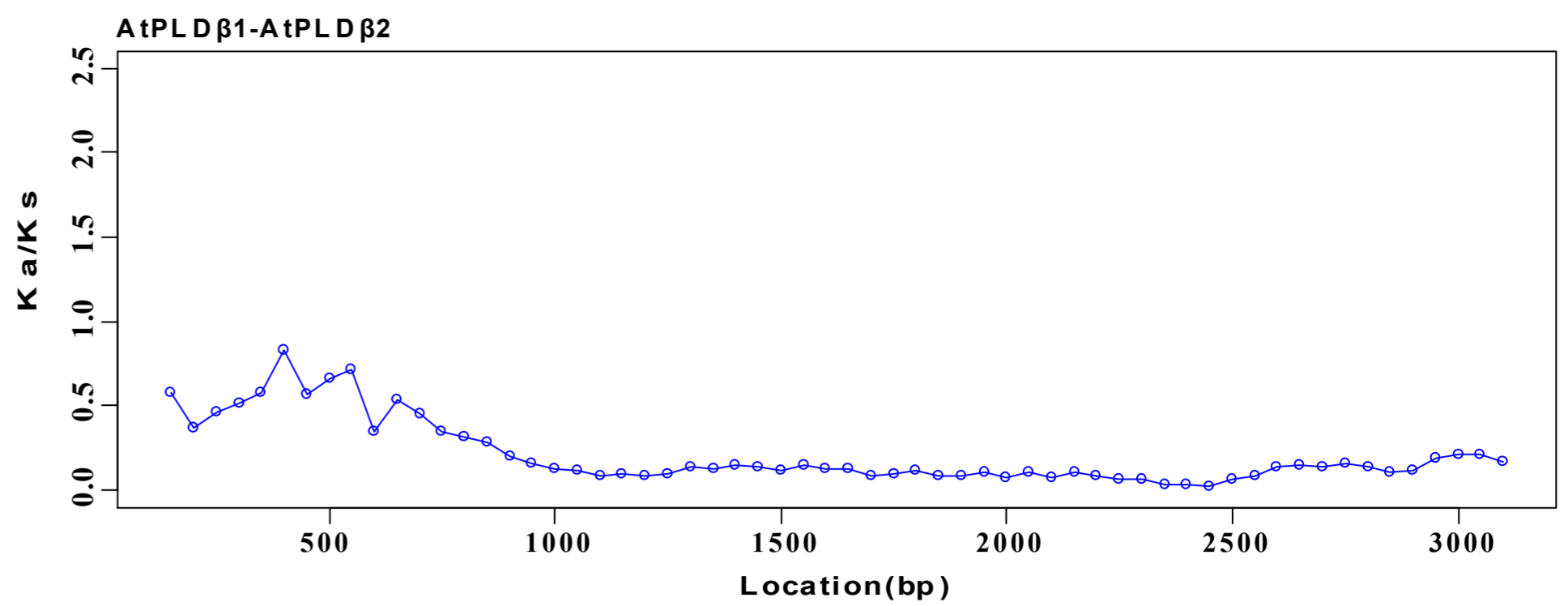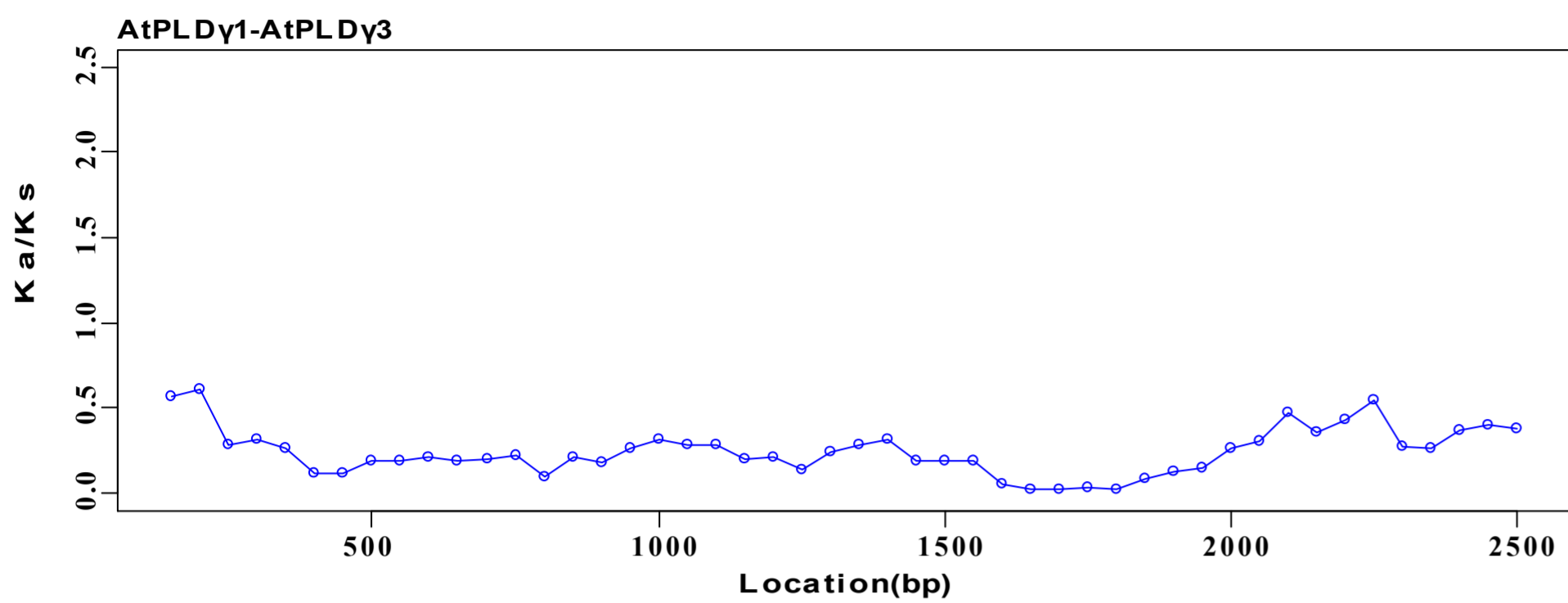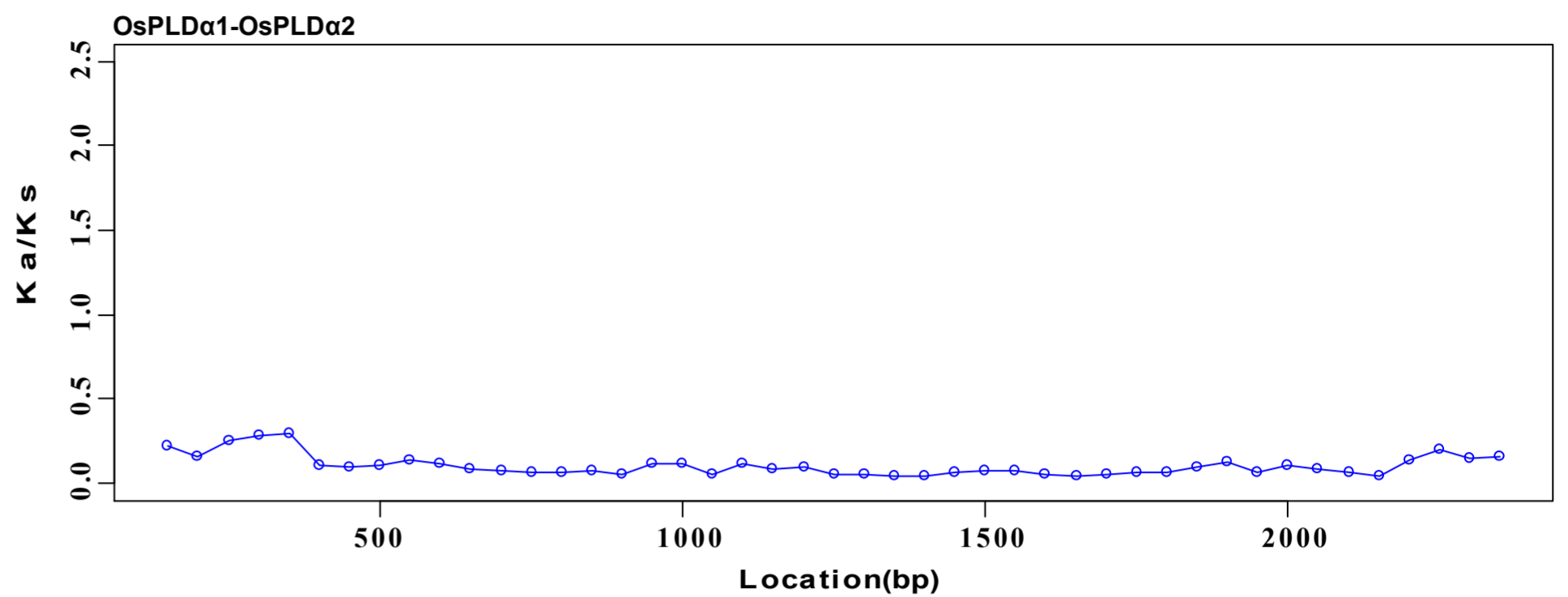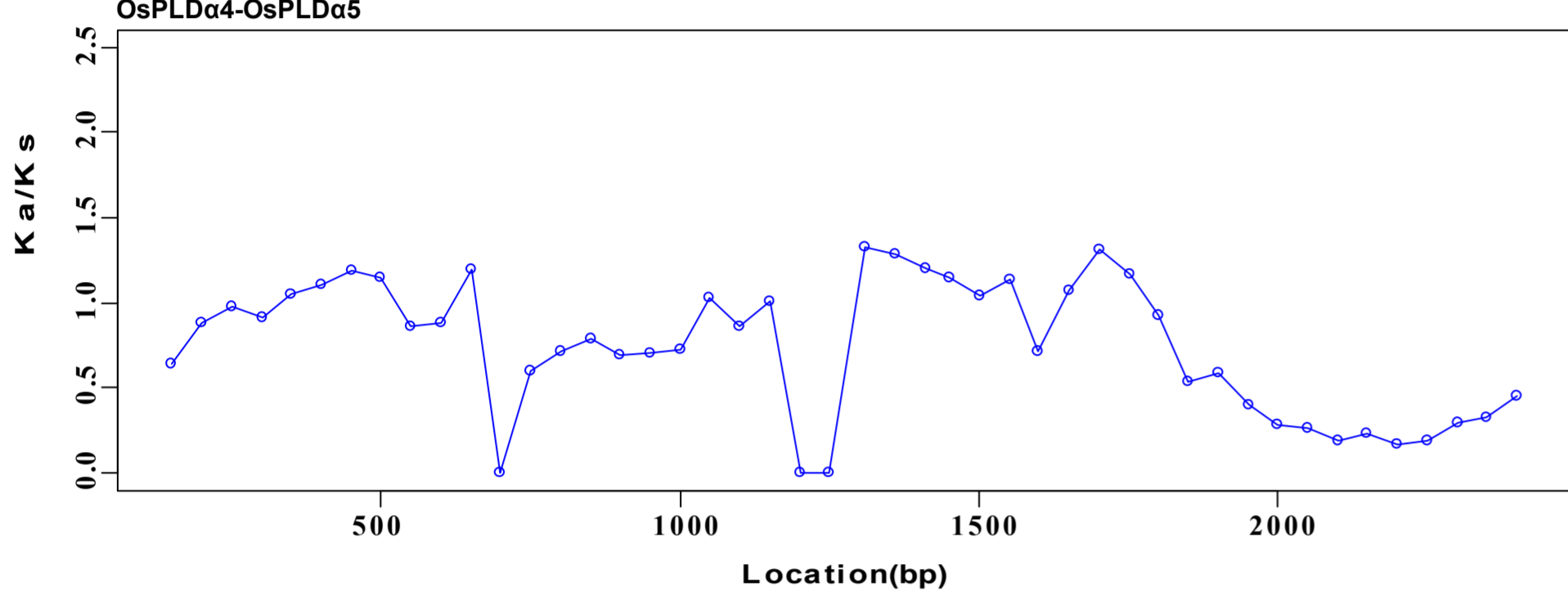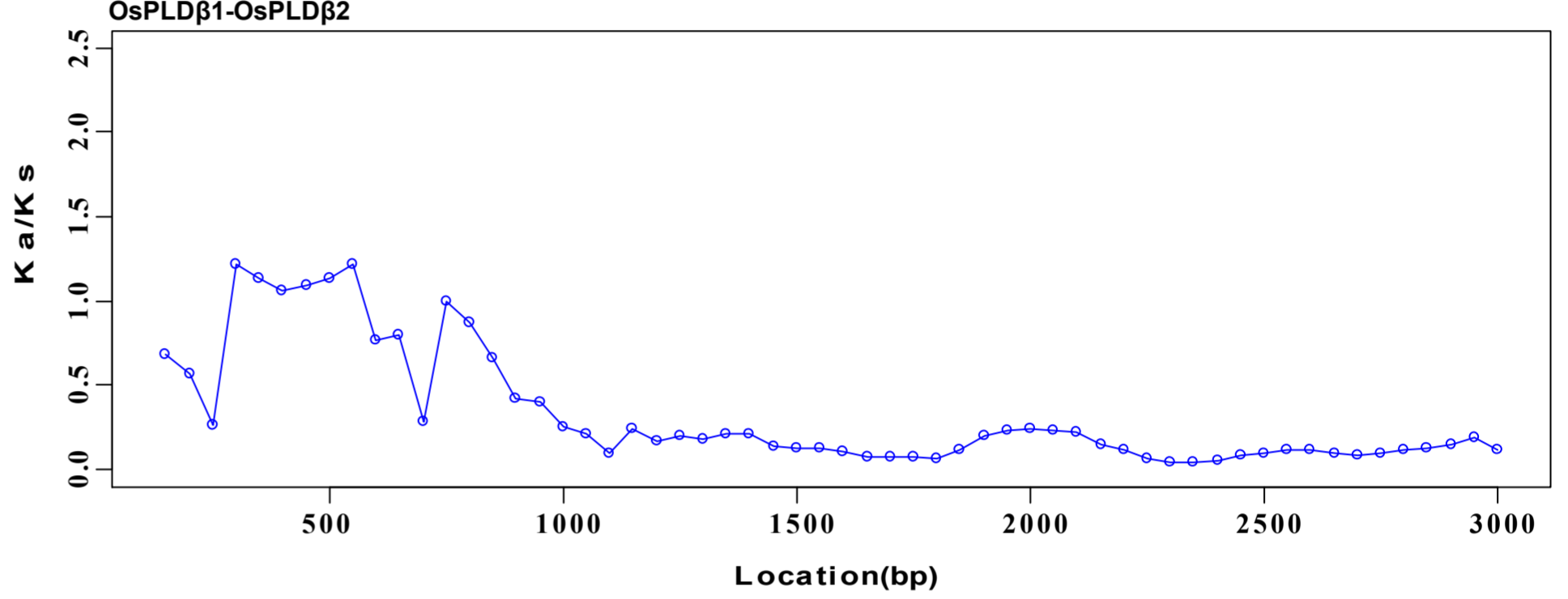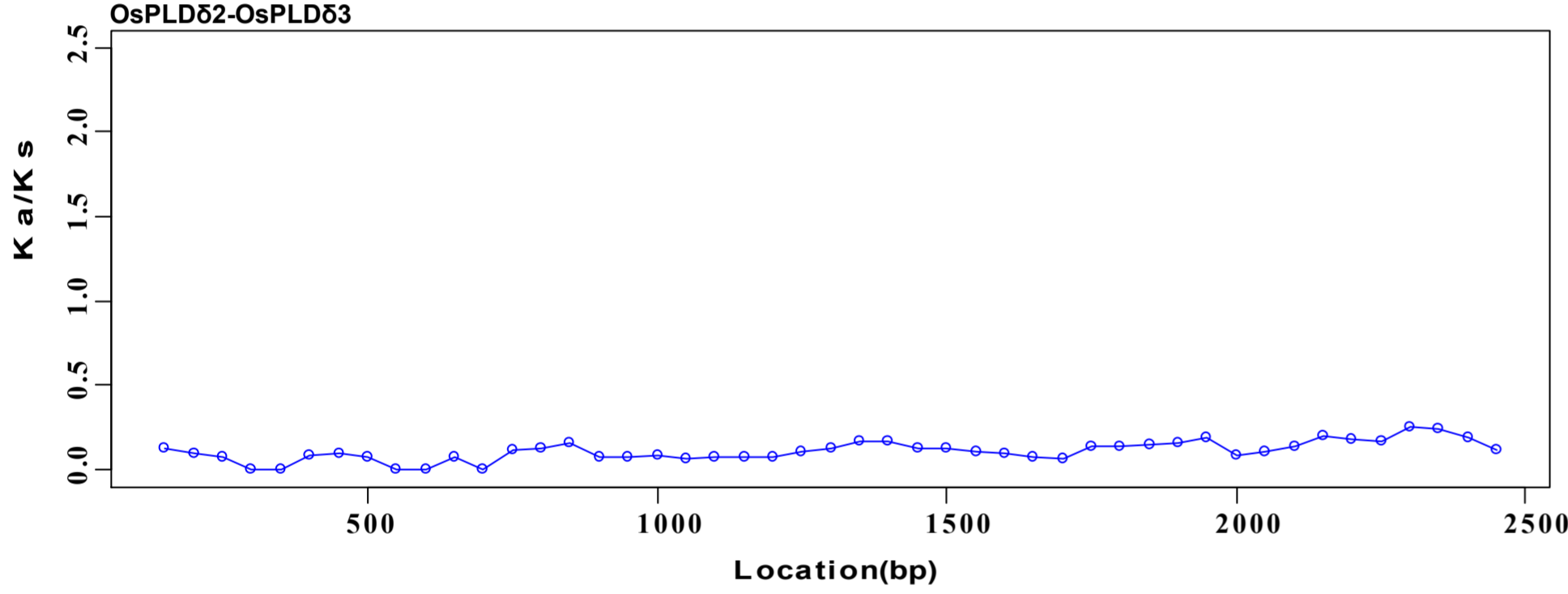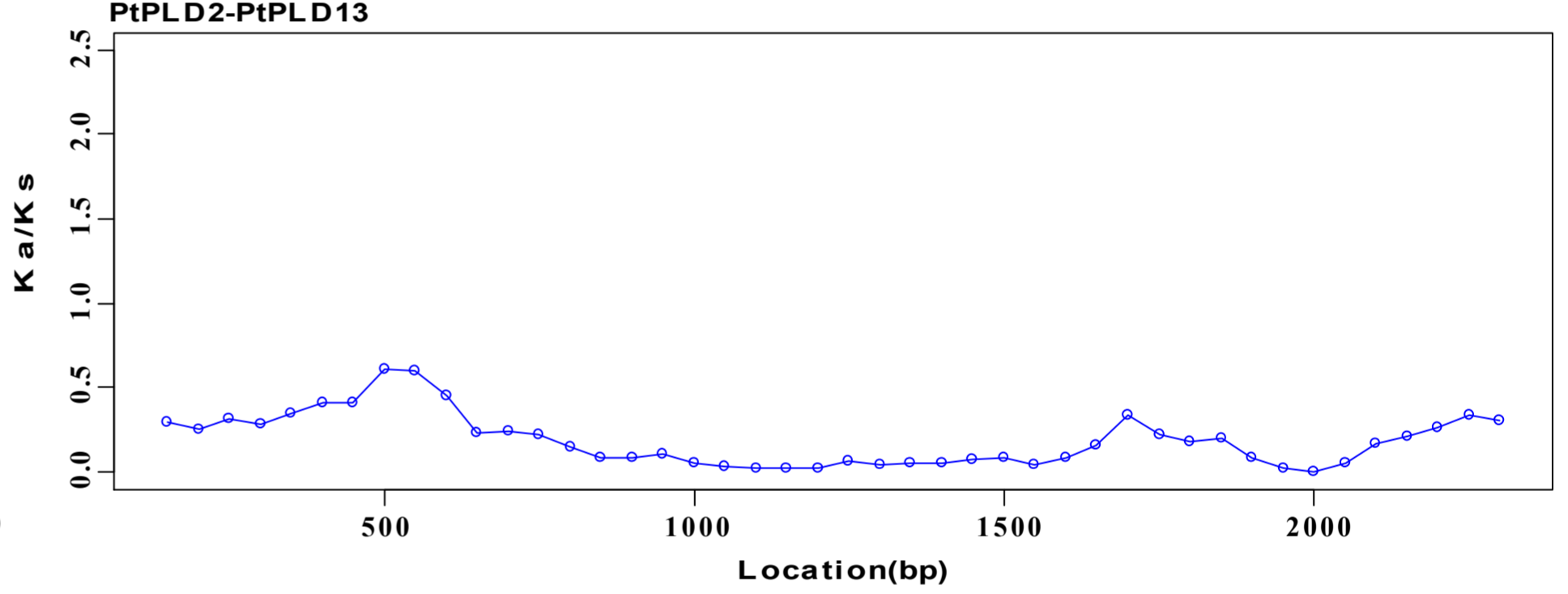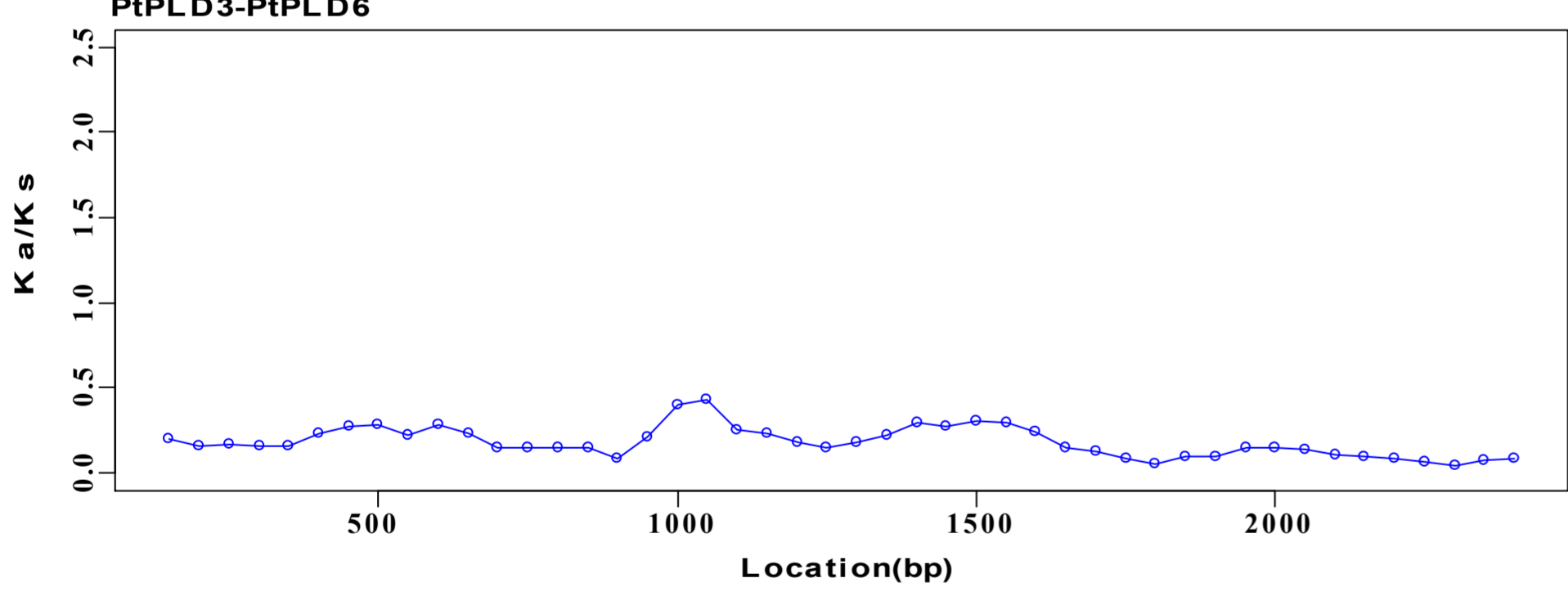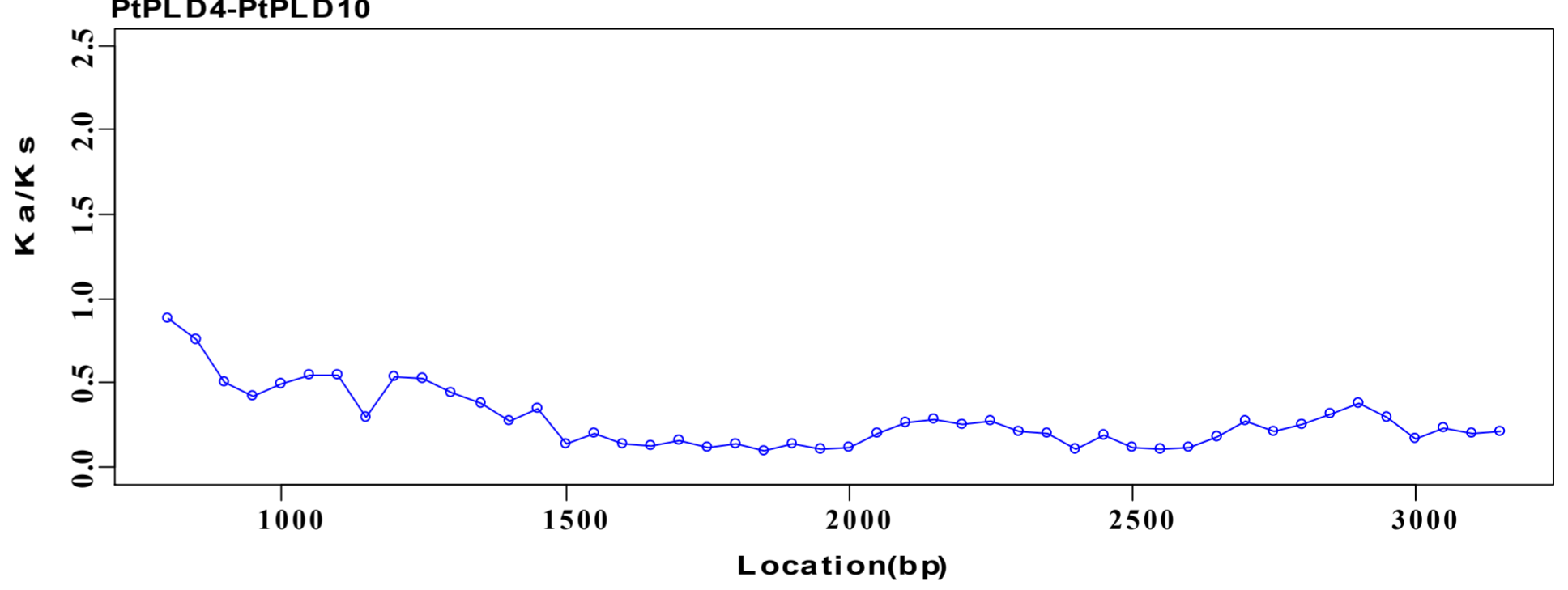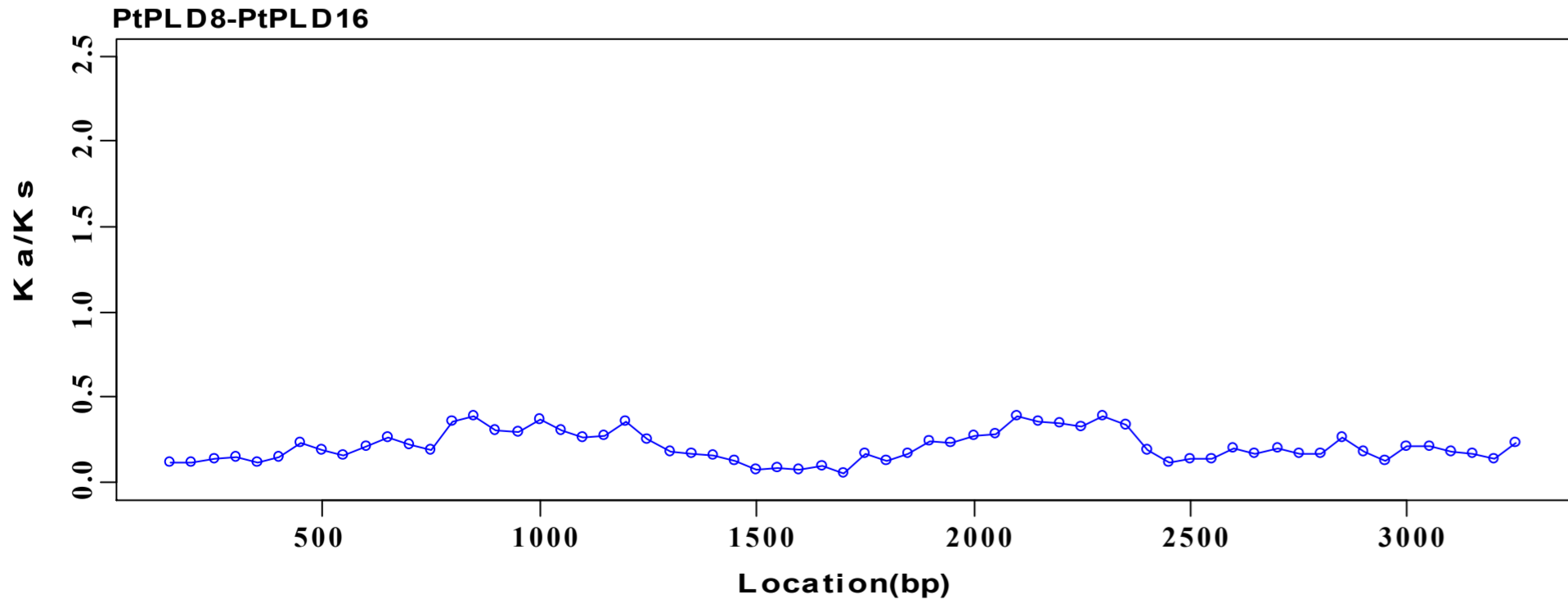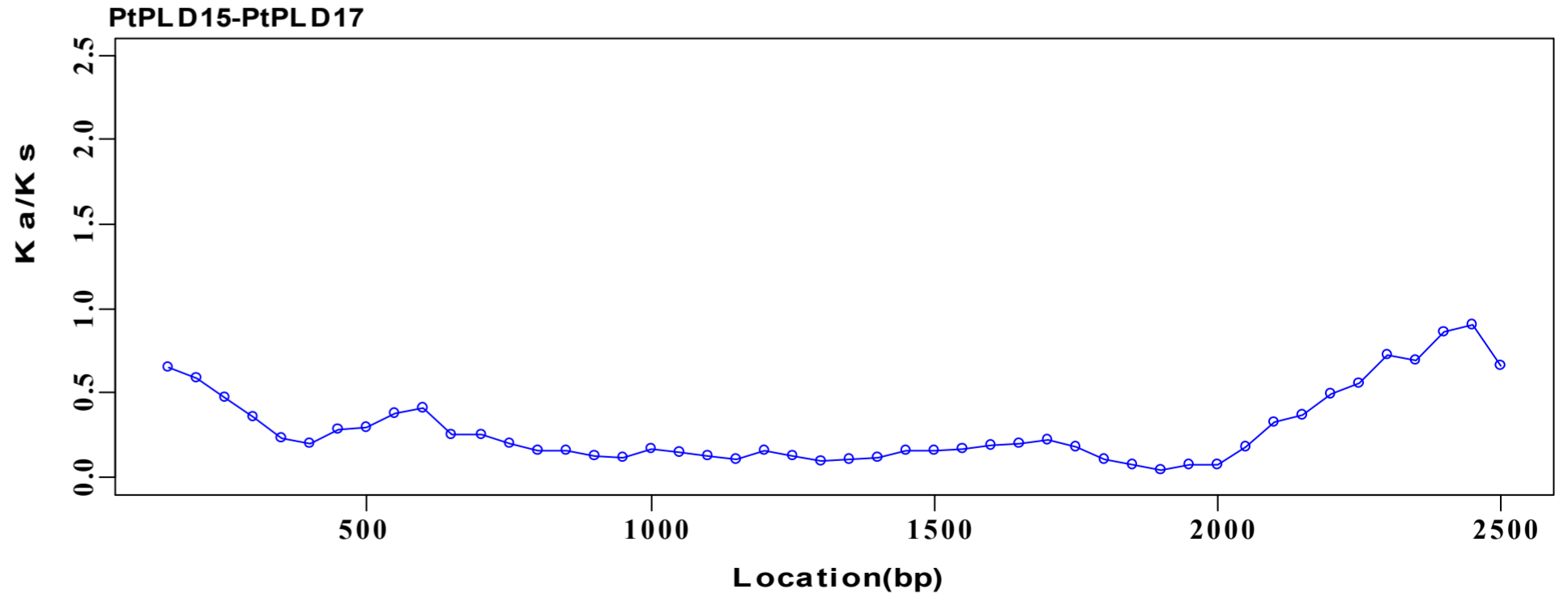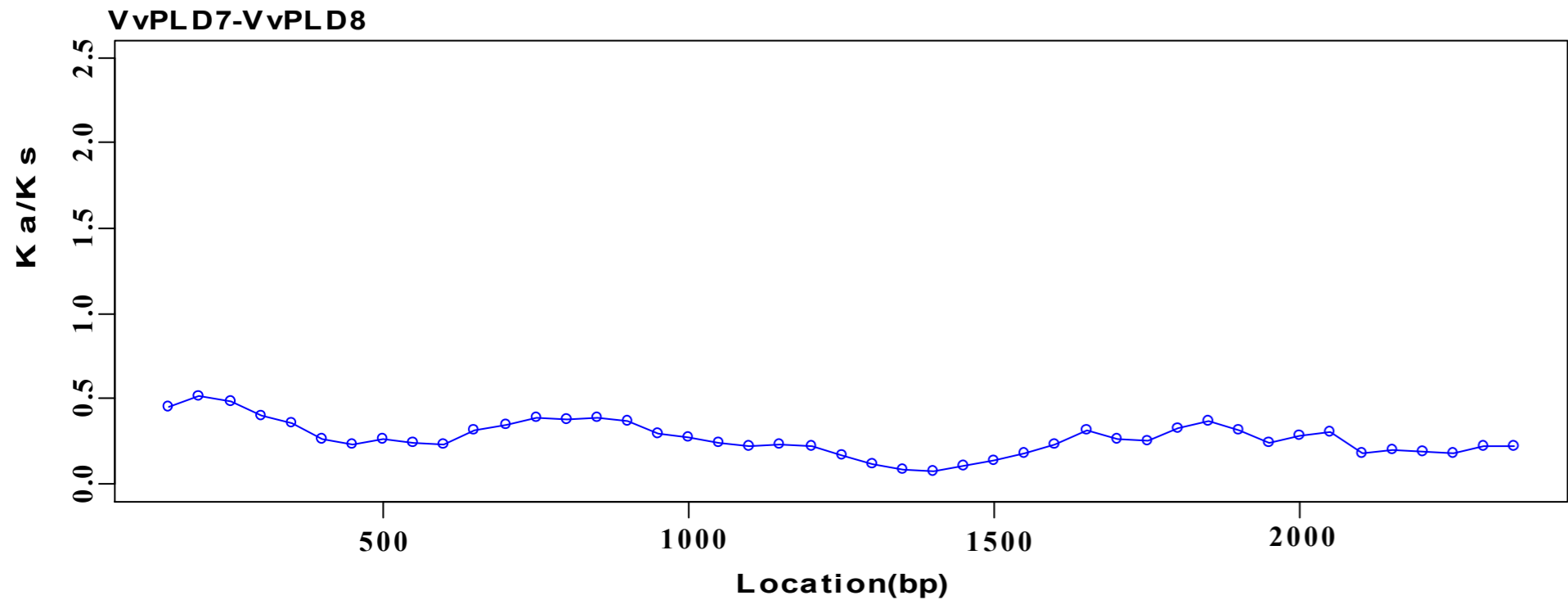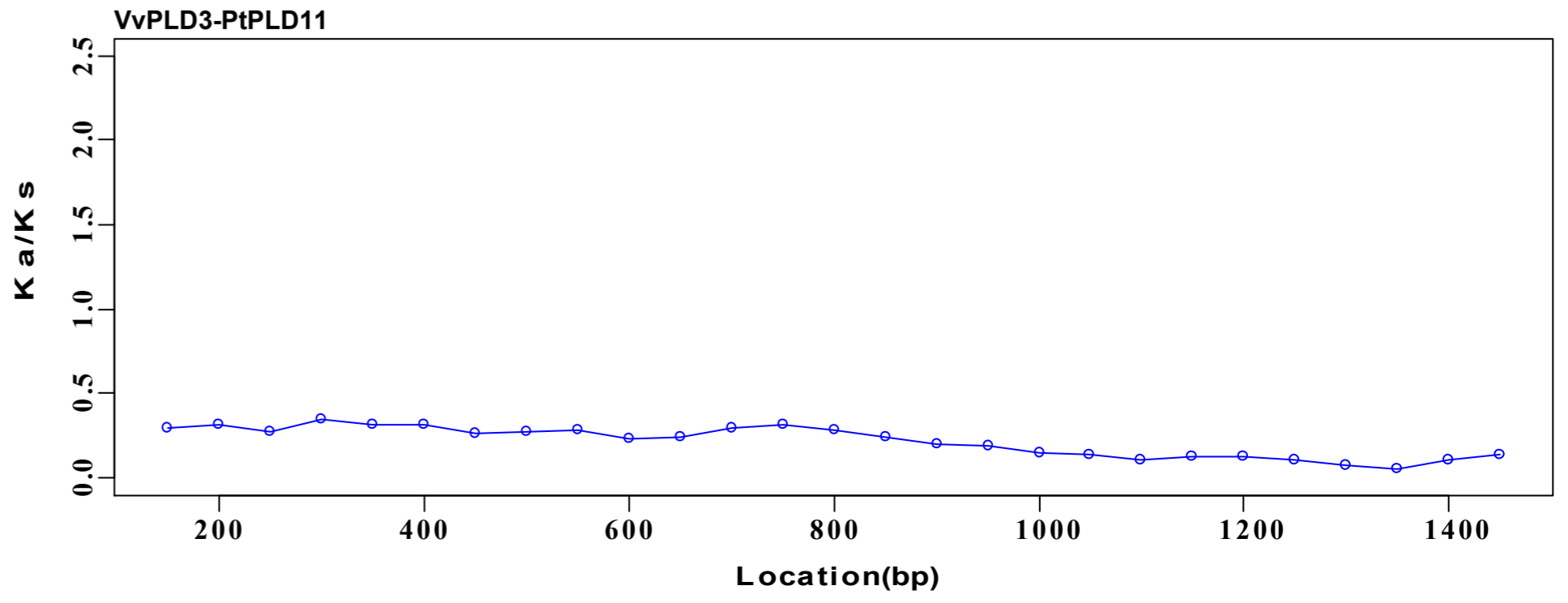

Supplement: Additional file 13 — Alignment of sequences of MEME motif 14 in PLD genes in Arabidopsis, rice, Poplar and Grape. Black and gray shadings indicate identical and conserved amino acid residues present in more than 50% of the aligned sequences, respectively. The colour bar and number above the sequence alignment represent MEME motifs. [file 1471-2229-10-117-S13.PDF]
