# Supplementary material for: Genome-wide and molecular evolution analyses of the phospholipase D gene family in Poplar and Grape
Source: BMC Plant Biol. 2010 Jun 18;10:117. doi: 10.1186/1471-2229-10-117 (PMC3095279; doi:10.1186/1471-2229-10-117)
Supplement: Additional file 3 — The ka/ks ratios for PLD paralogous genes in Arabidopsis, rice, Poplar and Grape. [file 1471-2229-10-117-S3.PDF]

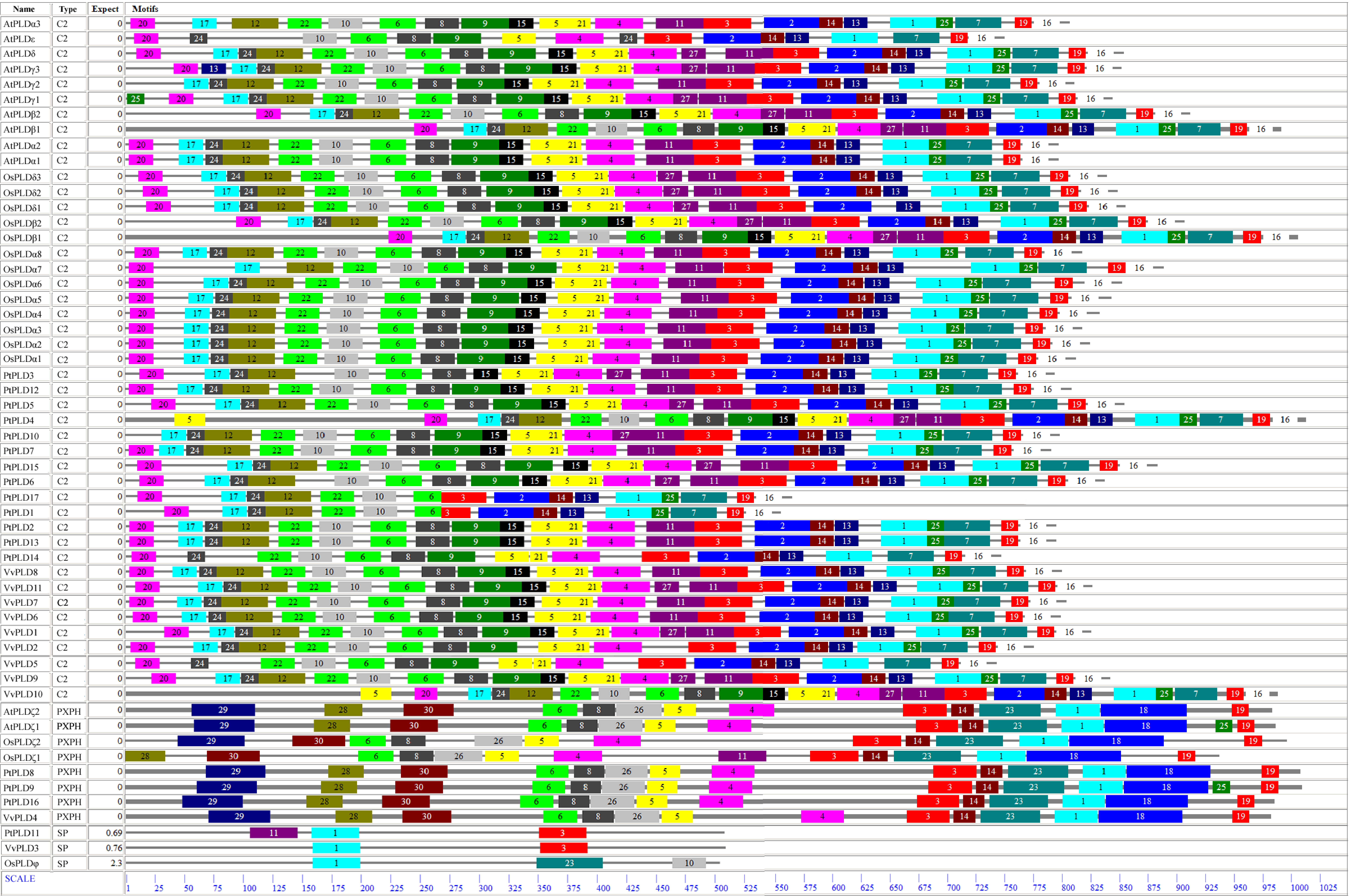

Name Type Expect Motifs
AtPLDα3 C2 0
AtPLDε C2 0
AtPLDδ C2 0
AtPLDγ3 C2 0
AtPLDγ2 C2 0
AtPLDγ1 C2 0
AtPLDβ2 C2 0
AtPLDβ1 C2 0
AtPLDα2 C2 0
AtPLDα1 C2 0
OsPLDδ3 C2 0
OsPLDδ2 C2 0
OsPLDδ1 C2 0
OsPLDβ2 C2 0
OsPLDβ1 C2 0
OsPLDα8 C2 0
OsPLDα7 C2 0
OsPLDα6 C2 0
OsPLDα5 C2 0
OsPLDα4 C2 0
OsPLDα3 C2 0
OsPLDα2 C2 0
OsPLDα1 C2 0
PtPLD3 C2 0
PtPLD12 C2 0
PtPLD5 C2 0
PtPLD4 C2 0
PtPLD10 C2 0
PtPLD7 C2 0
PtPLD15 C2 0
PtPLD6 C2 0
PtPLD17 C2 0
PtPLD1 C2 0
PtPLD2 C2 0
PtPLD13 C2 0
PtPLD14 C2 0
VvPLD8 C2 0
VvPLD11 C2 0
VvPLD7 C2 0
VvPLD6 C2 0
VvPLD1 C2 0
VvPLD2 C2 0
VvPLD5 C2 0
VvPLD9 C2 0
VvPLD10 C2 0
AtPLDζ2 PXPH 0
AtPLDζ1 PXPH 0
OsPLDζ2 PXPH 0
OsPLDζ1 PXPH 0
PtPLD8 PXPH 0
PtPLD9 PXPH 0
PtPLD16 PXPH 0
VvPLD4 PXPH 0
PtPLD11 SP 0.69
VvPLD3 SP 0.76
OsPLDφ SP 2.3
SCALE
1 25 50 75 100 125 150 175 200 225 250 275 300 325 350 375 400 425 450 475 500 525 550 575 600 625 650 675 700 725 750 775 800 825 850 875 900 925 950 975 1000 1025
