# Supplementary material for: Genome-wide and molecular evolution analyses of the phospholipase D gene family in Poplar and Grape
Source: BMC Plant Biol. 2010 Jun 18;10:117. doi: 10.1186/1471-2229-10-117 (PMC3095279; doi:10.1186/1471-2229-10-117)
Supplement: Additional file 4 — Parameter estimations and likelihood ratio tests for the site models in codeml. [file 1471-2229-10-117-S4.PDF]

Table S1. Function annotations of the motifs

| Motif | Length | Database Members Hit | InterPro Entry | Function Description |
|---|---|---|---|---|
| MOTIF1 | 41 | HMMPfam：PF00614<br>HMMPanther：PTHR18896<br>ProfileScan：PS50035 | IPR001736 | Phospholipase D<br>/Transphosphatidylase |
| MOTIF2 | 49 | HMMPanther:PTHR18896 | IPR015679 | Phospholipase D |
| MOTIF3 | 41 | HMMPanther:PTHR18896 | IPR015679 | Phospholipase D |
| MOTIF4 | 41 | Superfamily:SSF56024 | IPR015679 | Phospholipase D |
| MOTIF5 | 29 | HMMPanther:PTHR18896 | IPR015679 | Phospholipase D |
| MOTIF6 | 31 | HMMPanther:PTHR18896 | IPR015679 | Phospholipase D |
| MOTIF7 | 41 | HMMPanther:PTHR18896<br>HMMPfam:PF12357 | IPR015679 | Phospholipase D |
| MOTIF8 | 29 | HMMPanther:PTHR18896 | IPR015679 | Phospholipase D |
| MOTIF9 | 41 | HMMPanther:PTHR18896 | IPR015679 | Phospholipase D |
| MOTIF10 | 29 | HMMPanther:PTHR18896 | IPR015679 | Phospholipase D |
| MOTIF11 | 41 | HMMPanther:PTHR18896 | IPR015679 | Phospholipase D |
| MOTIF12 | 40 | HMMPanther:PTHR18896 | IPR015679 | Phospholipase D |
| MOTIF13 | 21 | HMMPanther:PTHR18896 | IPR015679 | Phospholipase D |
| MOTIF14 | 21 | HMMPanther:PTHR18896 | IPR015679 | Phospholipase D |
| MOTIF15 | 21 | HMMPanther:PTHR18896<br>HMMPfam:PF00614 | IPR001736 | Phospholipase D<br>/Transphosphatidylase |
| MOTIF16 | 23 | HMMPfam:PF12357<br>HMMPanther:PTHR18896 | IPR015679 | Phospholipase D |
| MOTIF17 | 21 | HMMPfam:PF00168<br>HMMPanther:PTHR18896 | IPR000008<br>IPR015679 | calcium-dependent membrane<br>targeting/Phospholipase D |
| MOTIF18 | 80 | HMMPanther:PTHR18896 | IPR015679 | Phospholipase D |
| MOTIF19 | 15 | HMMPfam:PF12357<br>HMMPanther:PTHR18896 | IPR015679 | Phospholipase D |
| MOTIF21 | 15 | HMMPanther:PTHR18896 | IPR015679 | Phospholipase D |
| MOTIF22 | 29 | HMMPanther:PTHR18896 | IPR015679 | Phospholipase D |
| MOTIF23 | 57 | HMMPanther:PTHR18896 | IPR015679 | Phospholipase D |
| MOTIF24 | 15 | HMMPanther:PTHR18896 | IPR015679 | Phospholipase D |
| MOTIF26 | 41 | HMMPanther:PTHR18896 | IPR015679 | Phospholipase D |
| MOTIF28 | 34 | HMMPanther:PTHR18896 | IPR015679 | Phospholipase D |
| MOTIF30 | 45 | HMMPanther:PTHR18896<br>SignalPHMM:SignalP | IPR015679 | Phospholipase D/Signal |
