# Supplementary material for: Genome-wide and molecular evolution analyses of the phospholipase D gene family in Poplar and Grape
Source: BMC Plant Biol. 2010 Jun 18;10:117. doi: 10.1186/1471-2229-10-117 (PMC3095279; doi:10.1186/1471-2229-10-117)
Supplement: Additional file 5 — Thirty putative motifs identified in all PLD gene family members in the four higher plants by MEME/MAST software. Different motifs are indicated by different colors. Names of all the members from different subfamilies and combined P values are shown on the left side of the figure and motif sizes are indicated at the bottom of the figure. [file 1471-2229-10-117-S5.PDF]

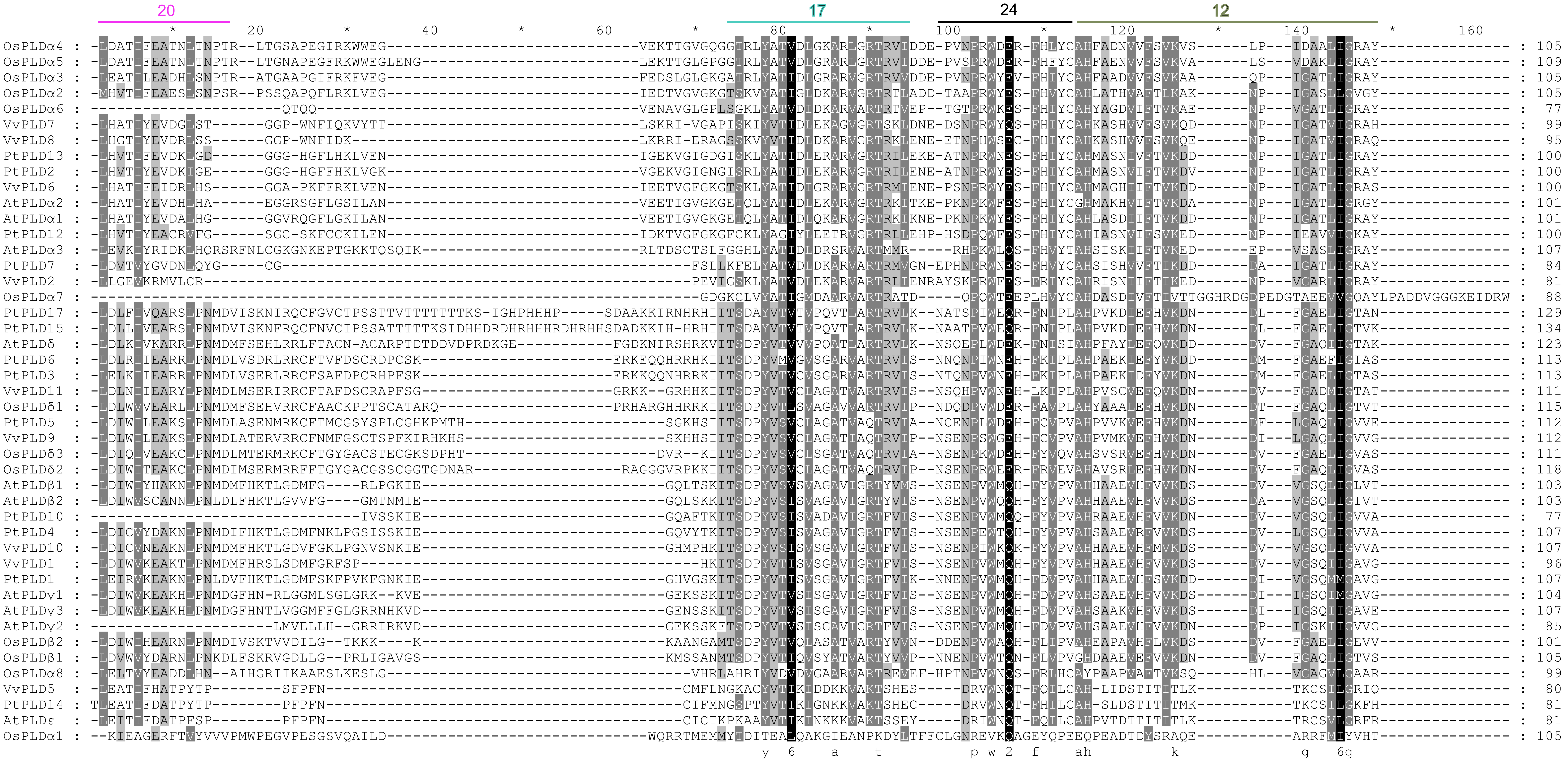

20
17
24
12
OsPLDα4
OsPLDα5
OsPLDα3
OsPLDα2
OsPLDα6
VvPLD7
VvPLD8
PtPLD13
PtPLD2
VvPLD6
AtPLDα2
AtPLDα1
PtPLD12
AtPLDα3
PtPLD7
VvPLD2
OsPLDα7
PtPLD17
PtPLD15
AtPLDδ
PtPLD6
PtPLD3
VvPLD11
OsPLDδ1
PtPLD5
VvPLD9
OsPLDδ3
OsPLDδ2
AtPLDβ1
AtPLDβ2
PtPLD10
PtPLD4
VvPLD10
VvPLD1
PtPLD1
AtPLDγ1
AtPLDγ3
AtPLDγ2
OsPLDβ2
OsPLDβ1
OsPLDα8
VvPLD5
PtPLD14
AtPLDε
OsPLDα1
