# Supplementary material for: Genome-wide and molecular evolution analyses of the phospholipase D gene family in Poplar and Grape
Source: BMC Plant Biol. 2010 Jun 18;10:117. doi: 10.1186/1471-2229-10-117 (PMC3095279; doi:10.1186/1471-2229-10-117)
Supplement: Additional file 6 — Function annotations of the motifs. Missing database hits of function annotations in motif 20, 25, 27 and 29 by InterProScan. [file 1471-2229-10-117-S6.PDF]

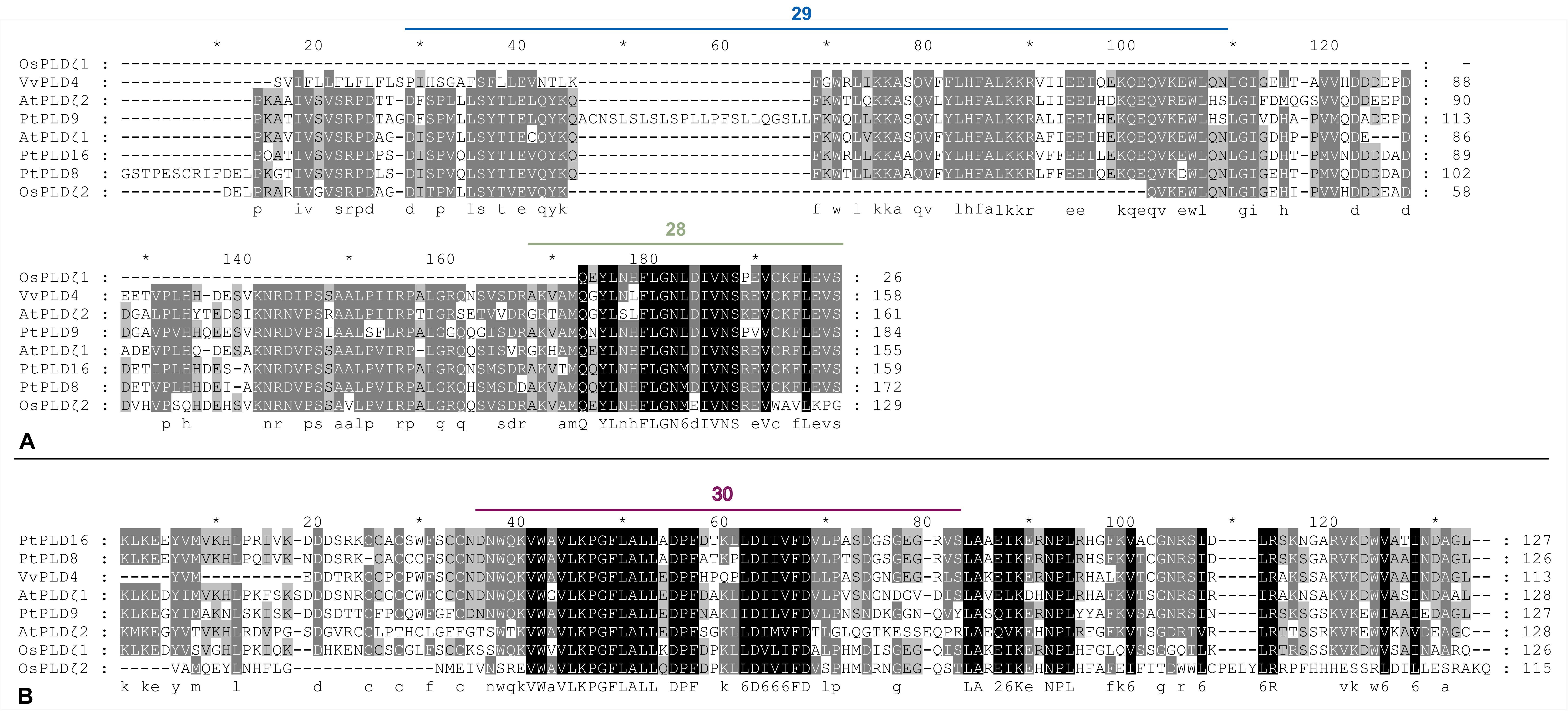
29
* 20 * 40 * 60 * 80 * 100 * 120
OsPLDζ1 : --- : -
VvPLD4 : ---------------SVIFLLFLFLFLSPIHSGAFSFLLEVNTLK-------------------------FGWRLIKKASQVFFLHFALKKRVIIEEIQEKQEQVKEWLQNIGIGEHT-AVVHDDDEPD : 88
AtPLDζ2 : -------------PKAAIVSVSRPDTT-DFSPLLLSYTLELQYKQ-------------------------FKWTLQKKASQVLYLHFALKKRLIIEELHDKQEQVREWLHSLGIFDMQGSVVQDDEEPD : 90
PtPLD9 : -------------PKATIVSVSRPDTAGDFSPMLLSYTIELQYKQACNSLSLSLSPLLPFSLLQGSLLFKWQLLKKASQVLYLHFALKKRALIEELHEKQEQVKEWLHSLGIVDHA-PVMQDADEPD : 113
AtPLDζ1 : -------------PKAVIVSVSRPDAG-DISPVLLSYTIECQYKQ-------------------------FKWQLVKKASQVFYLHFALKKRAFIEEIHEKQEQVKEWLQNLGIGDHP-PVVQDE---D : 86
PtPLD16 : -------------PQATIVSVSRPDPS-DISPVQLSYTIEVQYKQ-------------------------FKWRLLKKAAQVFYLHFALKKRVFFEEILEKQEQVKEWLQNLGIGDHT-PMVNDDDDAD : 89
PtPLD8 : GSTPESCRIFDELPKGTIVSVSRPDLS-DISPVQLSYTIEVQYKQ-------------------------FKWTLLKKAAQVFYLHFALKKRLFFEEIQEKQEQVKDWLQNLGIGEHT-PMVQDDDDAD : 102
OsPLDζ2 : ----------DELPRARIVGVSRPDAG-DITPMLLSYTVEVQYK--QVKEWLQNLGIGEHI-PVVHDDDEAD : 58
p iv srpd d p ls t e qyk f w l kka qv lhfalkkr ee kqeqv ewl gi h d d
28
* 140 * 160 * 180 *
OsPLDζ1 : --QEYLNHFLGNLDIVNSPEVCKFLEVS : 26
VvPLD4 : EETVPLHH-DESVKNRDIPSSAALPIIRPALGRQNSVSDRAKVAMQGYLNLFLGNLDIVNSREVCKFLEVS : 158
AtPLDζ2 : DGALPLHYTEDSIKNRNVPSRAALPIIRPTIGRSETVVDRGRTAMQGYLSLFLGNLDIVNSKEVCKFLEVS : 161
PtPLD9 : DGAVPVHHQEESVRNRDVPSIAALSFLRPALGGQQGISDRAKVAMQNYLNHFLGNLDIVNSPVVCKFLEVS : 184
AtPLDζ1 : ADEVPLHQ-DESAKNRDVPSSAALPVIRP-LGRQQSISVRGKHAMQEYLNHFLGNLDIVNSREVCRFLEVS : 155
PtPLD16 : DETIPLHHDES-AKNRDVPSSAALPVIRPALGRQNSMSDRAKVTMQQYLNHFLGNMDIVNSREVCKFLEVS : 159
PtPLD8 : DETVPLHHDEI-AKNRDVPSSAALPVIRPALGKQHSMSDDAKVAMQQYLNHFLGNMDIVNSREVCKFLEVS : 172
OsPLDζ2 : DVHVPSQHDEHSVKNRNVPSSAVLPVIRPALGRQQSVSDRAKVAMQEYLNHFLGNMEIVNSREVWAVLKPG : 129
p h nr ps aalp rp g q sdr amQ YLnhFLGN6dIVNS eVc fLevs
A
30
* 20 * 40 * 60 * 80 * 100 * 120 *
PtPLD16 : KLKEEYVMVKHLPRIVK-DDDSRKCCACSWFSCCNDNWQKVWAVLKPGFLALLADPFDTKLLDIIVFDVLPASDGSGEG-RVSLAAEIKERNPLRHGFKVACGNRSID----LRSKNGARVKDWVATINDAGL-- : 127
PtPLD8 : KLKEEYVMVKHLPQIVK-NDDSRK-CACCCFSCCNDNWQKVWAVLKPGFLALLADPFATKPLDIIVFDVLPTSDGSGEG-RVSLAAEIKERNPLRHSFKVTCGNRSID----LRSKSGARVKDWVAAINDAGL-- : 126
VvPLD4 : -----YVM----------EDDTRKCCPCPWFSCCNDNWQKVWAVLKPGFLALLEDPFHPQPLDIIVFDLLPASDGNGEG-RLSLAKEIKERNPLRHALKVTCGNRSIR----LRAKSSAKVKDWVAAINDAGL-- : 113
AtPLDζ1 : KLKEDYIMVKHLPKFSKSDDDSNRCCGCCWFCCCNDNWQKVWGVLKPGFLALLEDPFDAKLLDIIVFDVLPVSNGNDGV-DISLAVELKDHNPLRHAFKVTSGNRSIR----IRAKNSAKVKDWVASINDAAL-- : 128
PtPLD9 : KLKEGYIMAKNLSKISK-DDSDTTCFPCQWFGFCDNNWQKVWAVLKPGFLALLEDPFNAKIIDILVFDVLPNSNDKGGN-QVYLASQIKERNPLYYAFKVSAGNRSIN----LRSKSGSKVKEWIAAIEDAGL-- : 127
AtPLDζ2 : KMKEGYVTVKHLRDVPG-SDGVRCCLPTHCLGFFGTSWTKVWAVLKPGFLALLEDPFSGKLLDIMVFDTLGLQGTKESSEQPRLAEQVKEHNPLRFGFKVTSGDRTVR----LRTTSSRKVKEWVKAVDEAGC-- : 128
OsPLDζ1 : KLKEDYVSVGHLPKIQK--DHKENCCSCGLFSCCKSSWQKVWVVLKPGFLALLKDPFDPKLLDVLIFDALPHMDISGEG-QISLAKEIKERNPLHFGLQVSSGGQTLK----LRTRSSSKVKEWVSAINAARQ-- : 126
OsPLDζ2 : -----VAMQEYLNHFLG--------------NMEIVNSREVWAVLKPGFLALLQDPFDPKLLDIVIFDVSPHMDRNGEG-QSTLAREIKEHNPLHFAEEIFITDWWLCPELYLRRPFHHESSRLDILLESRAKQ : 115
k ke y m l d c c f c nwqkVWaVLKPGFLALL DPF k 6D666FD lp g LA 26Ke NPL fk6 g r 6 6R vk w6 6 a
B
