# Supplementary material for: Genome-wide and molecular evolution analyses of the phospholipase D gene family in Poplar and Grape
Source: BMC Plant Biol. 2010 Jun 18;10:117. doi: 10.1186/1471-2229-10-117 (PMC3095279; doi:10.1186/1471-2229-10-117)
Supplement: Additional file 7 — Alignment of sequences of C2 domain of PLD genes in Arabidopsis, rice, Poplar and Grape. Black and gray shading indicate identical and conserved amino acid residues present in more than 50% of the aligned sequences, respectively. The colour bars and numbers above the sequence alignment represent MEME motifs. [file 1471-2229-10-117-S7.PDF]

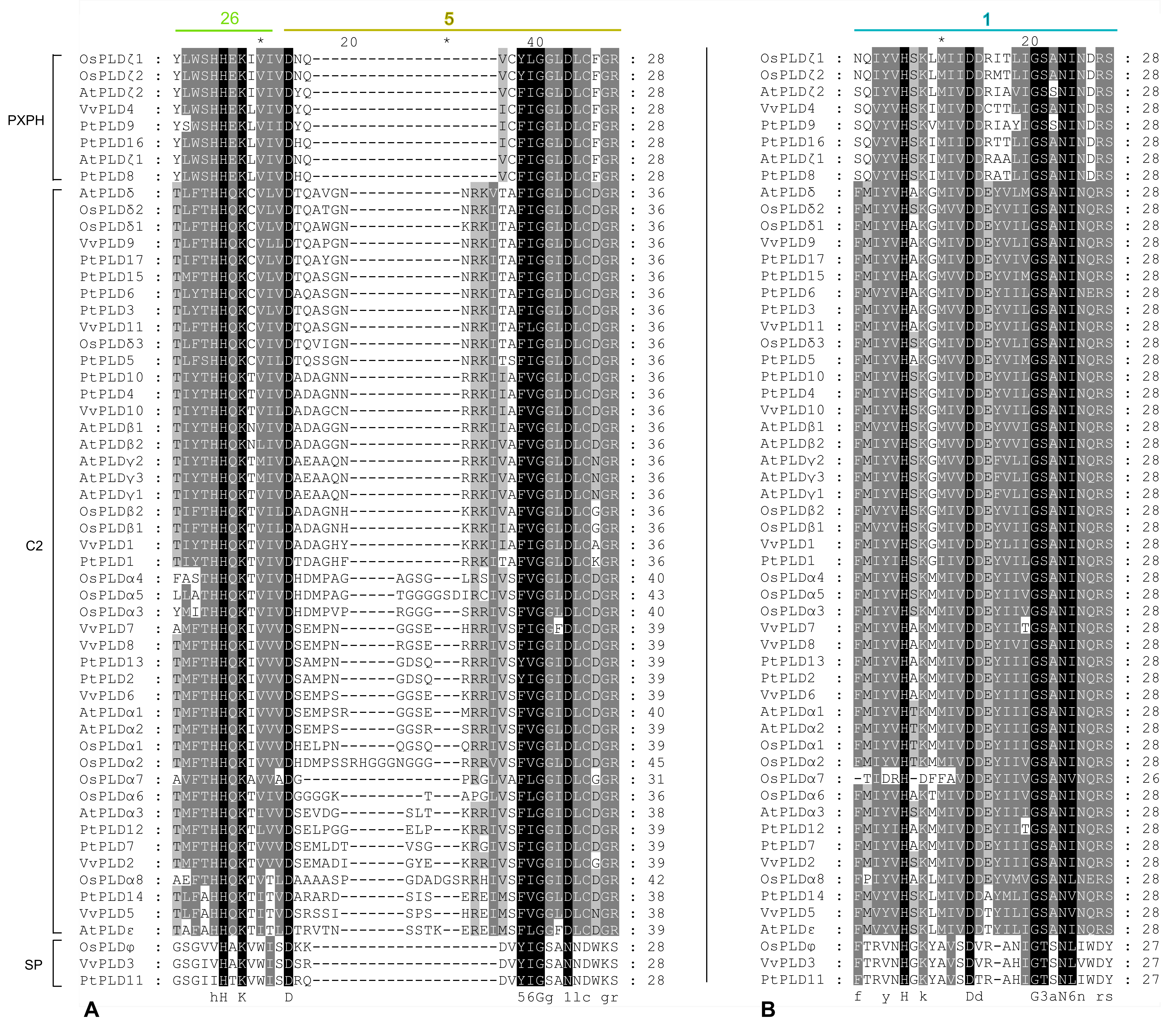

26
5
* 20 * 40
PXPH
OsPLDζ1 : YLWSHHEKIVIVDNQ--------------------VCYLGGLDLCFGR : 28
OsPLDζ2 : YLWSHHEKIVIVDNQ--------------------VCYIGGLDLCFGR : 28
AtPLDζ2 : YLWSHHEKIVIVDYQ--------------------VCFIGGLDLCFGR : 28
VvPLD4 : YLWSHHEKLVIVDYQ--------------------ICFIGGLDLCFGR : 28
PtPLD9 : YSWSHHEKLVIIDYQ--------------------ICFIGGLDLCFGR : 28
PtPLD16 : YLWSHHEKLVIVDHQ--------------------ICFIGGLDLCFGR : 28
AtPLDζ1 : YLWSHHEKLVIVDNQ--------------------VCFIGGLDLCFGR : 28
PtPLD8 : YLWSHHEKLVIVDHQ--------------------VCFIGGLDLCFGR : 28
C2
AtPLDδ : TLFTHHQKCVLVDTQAVGN------------NRKVTAFIGGLDLCDGR : 36
OsPLDδ2 : TLFTHHQKCVLVDTQATGN------------NRKITAFIGGLDLCDGR : 36
OsPLDδ1 : TLFTHHQKCVLVDTQAWGN------------KRKITAFIGGLDLCDGR : 36
VvPLD9 : TLFTHHQKCVLLDTQAPGN------------NRKITAFIGGLDLCDGR : 36
PtPLD17 : TIFTHHQKCVLVDTQAYGN------------NRKITAFIGGIDLCDGR : 36
PtPLD15 : TMFTHHQKCVLVDTQASGN------------NRKITAFIGGIDLCDGR : 36
PtPLD6 : TLYTHHQKCVIVDAQASGN------------NRKITAFIGGLDLCDGR : 36
PtPLD3 : TLYTHHQKCVLVDTQASGN------------NRKITAFIGGLDLCDGR : 36
VvPLD11 : TLFTHHQKCVIVDTQASGN------------NRKITAFLGGLDLCDGR : 36
OsPLDδ3 : TLFTHHQKCVIVDTQVIGN------------NRKITAFIGGLDLCDGR : 36
PtPLD5 : TLFSHHQKCVILDTQSSGN------------NRKITSFIGGLDLCDGR : 36
PtPLD10 : TIYTHHQKTVIVDADAGNN------------RRKIIAFVGGLDLCDGR : 36
PtPLD4 : TIYTHHQKTVIVDADAGNN------------RRKIIAFVGGLDLCDGR : 36
VvPLD10 : TIYTHHQKTVILDADAGCN------------RRKIIAFVGGLDLCDGR : 36
AtPLDβ1 : TIYTHHQKNVIVDADAGGN------------RRKIIAFVGGLDLCDGR : 36
AtPLDβ2 : TIYTHHQKNLIVDADAGGN------------RRKIVAFVGGLDLCDGR : 36
AtPLDγ2 : TIYTHHQKTMIVDAEAAQN------------RRKIVAFVGGLDLCNGR : 36
AtPLDγ3 : TIYTHHQKTMIVDAEAAQN------------RRKIVAFVGGLDLCNGR : 36
AtPLDγ1 : TIYTHHQKTVIVDAEAAQN------------RRKIVAFVGGLDLCNGR : 36
OsPLDβ2 : TIFTHHQKTVILDADAGNH------------KRKIVAFVGGLDLCGGR : 36
OsPLDβ1 : TIFTHHQKTVILDADAGNH------------KRKIIAFVGGLDLCGGR : 36
VvPLD1 : TIYTHHQKTVIVDADAGHY------------KRKIIAFIGGLDLCAGR : 36
PtPLD1 : TIYTHHQKTVIVDTDAGHF------------RRKITAFVGGLDLCKGR : 36
OsPLDα4 : FASTHHQKTVIVDHDMPAG-----AGSG---LRSIVSFVGGLDLCDGR : 40
OsPLDα5 : LLATHHQKTVIVDHDMPAG-----TGGGGSDIRCIVSFVGGLDLCDGR : 43
OsPLDα3 : YMITHHQKTVIVDHDMPVP-----RGGG---SRRIVSFVGGLDLCDGR : 40
VvPLD7 : AMFTHHQKIVVVDSEMPN------GGSE---HRRIVSFIGGFDLCDGR : 39
VvPLD8 : TMFTHHQKIVVVDSEMPN------RGSE---RRRIVSFIGGIDLCDGR : 39
PtPLD13 : TMFTHHQKIVVVDSAMPN------GDSQ---RRRIVSYVGGIDLCDGR : 39
PtPLD2 : TMFTHHQKIVVVDSAMPN------GDSQ---RRRIVSYIGGIDLCDGR : 39
VvPLD6 : TMFTHHQKIVVVDSEMPS------GGSE---KRRIVSFVGGIDLCDGR : 39
AtPLDα1 : TMFTHHQKIVVVDSEMPSR-----GGSE---MRRIVSFVGGIDLCDGR : 40
AtPLDα2 : TMFTHHQKIVVVDSEMPS------GGSR---SRRIVSFVGGLDLCDGR : 39
OsPLDα1 : TMFTHHQKIVVVDHELPN------QGSQ---QRRIVSFVGGLDLCDGR : 39
OsPLDα2 : TMFTHHQKIVVVDHDMPSSRHGGGNGGG---RRRVVSFVGGLDLCDGR : 45
OsPLDα7 : AVFTHHQKAVVADG-----------------PRGLVAFLGGIDLCGGR : 31
OsPLDα6 : TMFTHHQKTVIVDGGGGK---------T---APGLVSFLGGIDLCDGR : 36
AtPLDα3 : TMFTHHQKTIVVDSEVDG-------SLT---KRRIVSFLGGIDLCDGR : 38
PtPLD12 : TMFTHHQKTLVVDSELPGG------ELP---KRRIVSFIGGIDLCDGR : 39
PtPLD7 : TMFTHHQKTVVVDSEMLDT------VSG---KRGIVSFIGGIDLCDGR : 39
VvPLD2 : TMFTHHQKTVVVDSEMADI------GYE---KRRIVSFVGGIDLCGGR : 39
OsPLDα8 : AEFTHHQKTVTLDAAAASP------GDADGSRRHIVSFIGGIDLCDGR : 42
PtPLD14 : TLFAHHQKTITVDARARD-------SIS---EREIMSFVGGLDLCDGR : 38
VvPLD5 : TLFAHHQKTITVDSRSSI-------SPS---HREIMSFVGGLDLCNGR : 38
AtPLDε : TAFAHHQKTITLDTRVTN-------SSTK--EREIMSFLGGFDLCDGR : 39
SP
OsPLDφ : GSGVVHAKVWISDKK--------------------DVYIGSANNDWKS : 28
VvPLD3 : GSGIVHAKVWISDSR--------------------DVYIGSANNDWKS : 28
PtPLD11 : GSGIIHTKVWISDRQ--------------------DVYIGSANNDWKS : 28
hH K D 56Gg 1lc gr
A
1
* 20
OsPLDζ1 : NQIYVHSKLMIIDDRITLIGSANINDRS : 28
OsPLDζ2 : NQIYVHSKLMIIDDRMTLIGSANINDRS : 28
AtPLDζ2 : SQIYVHSKLMIVDDRIAVIGSSNINDRS : 28
VvPLD4 : SQVYVHSKIMIVDDCTTLIGSANINDRS : 28
PtPLD9 : SQVYVHSKVMIVDDRIAYIGSSNINDRS : 28
PtPLD16 : SQVYVHSKIMIIDDRTTLIGSANINDRS : 28
AtPLDζ1 : SQVYVHSKIMIVDDRAALIGSANINDRS : 28
PtPLD8 : SQVYVHSKIMIVDDRATLIGSANINDRS : 28
AtPLDδ : FMIYVHAKGMIVDDEYVLMGSANINQRS : 28
OsPLDδ2 : FMIYVHSKGMVVDDEYVIIGSANINQRS : 28
OsPLDδ1 : FMIYVHAKGMIVDDEYVILGSANINQRS : 28
VvPLD9 : FMIYVHAKGMIVDDEYVLIGSANINQRS : 28
PtPLD17 : FMIYVHAKGMIVDDEYVIVGSANINQRS : 28
PtPLD15 : FMIYVHAKGMVVDDEYVIMGSANINQRS : 28
PtPLD6 : FMVYVHAKGMIVDDEYIILGSANINERS : 28
PtPLD3 : FMIYVHAKGMVVDDEYVILGSANINQRS : 28
VvPLD11 : FMIYVHAKGMIVDDEYVILGSANINQRS : 28
OsPLDδ3 : FMIYVHSKGMIVDDEYVLIGSANINQRS : 28
PtPLD5 : FMIYVHAKGMVVDDEYVIMGSANINQRS : 28
PtPLD10 : FMIYVHSKGMIVDDEYVILGSANINQRS : 28
PtPLD4 : FMIYVHSKGMIVDDEYVILGSANINQRS : 28
VvPLD10 : FMIYVHSKGMIVDDEYVILGSANINQRS : 28
AtPLDβ1 : FMVYVHSKGMVVDDEYVVIGSANINQRS : 28
AtPLDβ2 : FMIYVHSKGMVVDDEYVVIGSANINQRS : 28
AtPLDγ2 : FMIYVHSKGMVVDDEFVLIGSANINQRS : 28
AtPLDγ3 : FMIYVHSKGMVVDDEFVLIGSANINQRS : 28
AtPLDγ1 : FMIYVHSKGMVVDDEFVLIGSANINQRS : 28
OsPLDβ2 : FMVYVHSKGMIVDDEYVIIGSANINQRS : 28
OsPLDβ1 : FMVYVHSKGMIVDDEYVIIGSANINQRS : 28
VvPLD1 : FMIYVHSKGMIVDDEYLIIGSANINQRS : 28
PtPLD1 : FMIYIHSKGIIVDDEYVILGSANINQRS : 28
OsPLDα4 : FMIYVHSKMMIVDDEYIIVGSANINQRS : 28
OsPLDα5 : FMIYVHSKMMIVDDEYIIVGSANINQRS : 28
OsPLDα3 : FMIYVHSKMMIVDDEYIIVGSANINQRS : 28
VvPLD7 : FMIYVHAKMMIVDDEYIITGSANINQRS : 28
VvPLD8 : FMIYVHAKMMIVDDEYIVIGSANINQRS : 28
PtPLD13 : FMIYVHAKMMIVDDEYIIIGSANINQRS : 28
PtPLD2 : FMIYVHAKMMIVDDEYIIIGSANINQRS : 28
VvPLD6 : FMIYVHAKMMIVDDEYIIIGSANINQRS : 28
AtPLDα1 : FMIYVHTKMMIVDDEYIIIGSANINQRS : 28
AtPLDα2 : FMIYVHTKMMIVDDEYIIIGSANINQRS : 28
OsPLDα1 : FMIYVHTKMMIVDDEYIIIGSANINQRS : 28
OsPLDα2 : FMIYVHTKMMIVDDEYIIVGSANINQRS : 28
OsPLDα7 : -TIDRH-DFFAVDDEYIIVGSANVNQRS : 26
OsPLDα6 : FMIYVHAKTMIVDDEYIIVGSANINQRS : 28
AtPLDα3 : FMIYVHSKMMIVDDEYIIIGSANINQRS : 28
PtPLD12 : FMIYIHAKMMIVDDEYIITGSANINQRS : 28
PtPLD7 : FMIYVHAKMMIVDDEYIIIGSANINQRS : 28
VvPLD2 : FMIYVHSKMMIVDDEYIIIGSANINQRS : 28
OsPLDα8 : FPIYVHAKLMIVDDEYVMVGSANLNERS : 28
PtPLD14 : FMVYVHSKLMIVDDAYMLIGSANVNQRS : 28
VvPLD5 : FMVYVHSKLMIVDDTYILIGSANVNQRS : 28
AtPLDε : FMVYVHSKLMIVDDTYILIGSANINQRS : 28
OsPLDφ : FTRVNHGKYAVSDVR-ANIGTSNLIWDY : 27
VvPLD3 : FTRVNHGKYAVSDVR-AHIGTSNLVWDY : 27
PtPLD11 : FTRVNHGKYAVSDTR-AHIGTSNLIWDY : 27
f y H k Dd G3aN6n rs
B
