# Supplementary material for: Genome-wide and molecular evolution analyses of the phospholipase D gene family in Poplar and Grape
Source: BMC Plant Biol. 2010 Jun 18;10:117. doi: 10.1186/1471-2229-10-117 (PMC3095279; doi:10.1186/1471-2229-10-117)
Supplement: Additional file 8 — Alignment of sequences of PX domain (A) and PH domain (B) of PLD genes in Arabidopsis, rice, Poplar and Grape. Black and gray shading indicate identical and conserved amino acid residues present in more than 50% of the aligned sequences, respectively. The colour bar and numbers above the sequence alignment represent MEME motifs. [file 1471-2229-10-117-S8.PDF]

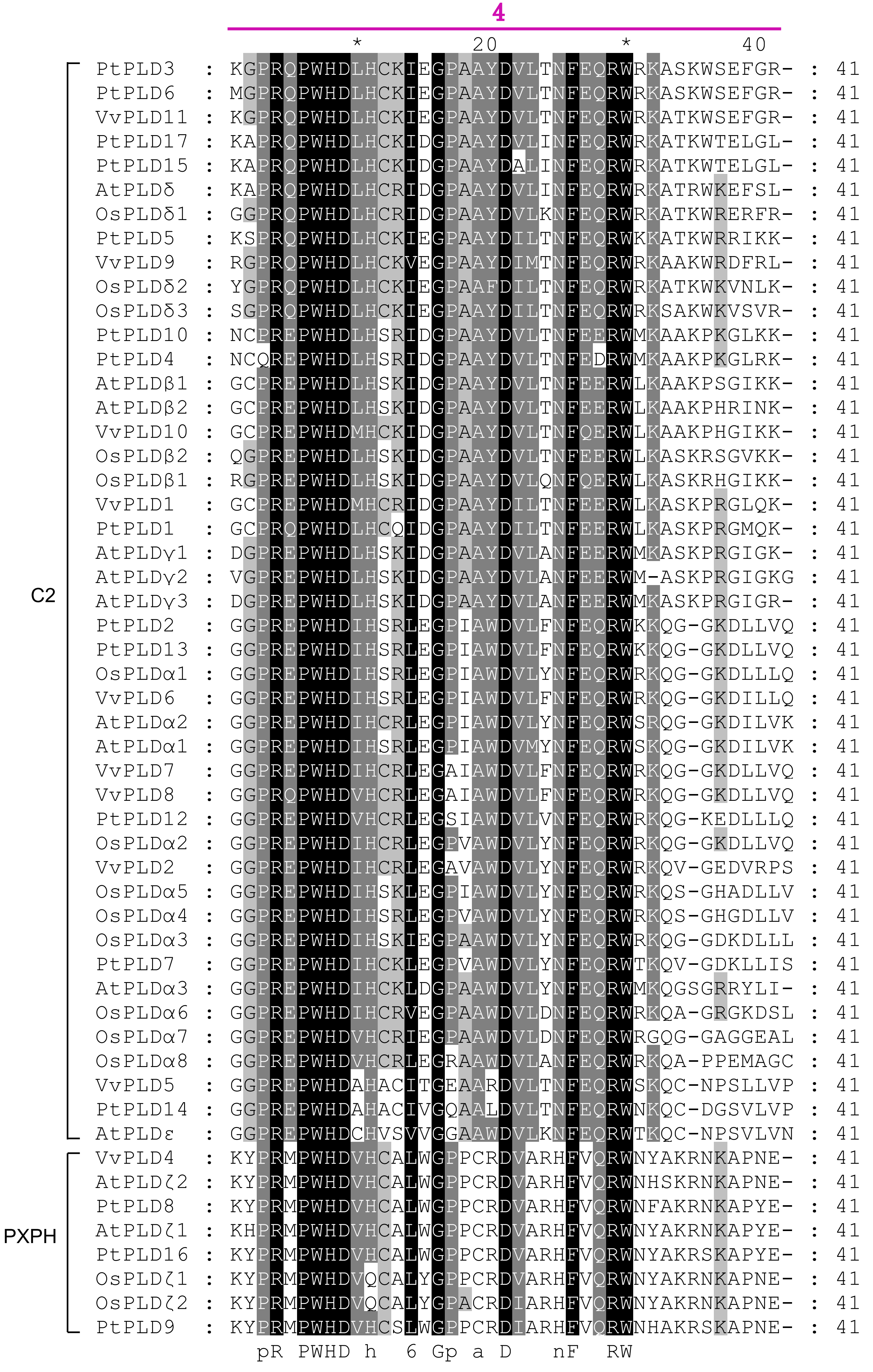
4
* 20 * 40
C2
PtPLD3 : KGPRQPWHDLHCKIEGPAAYDVLTNFEQRWRKASKWSEFGR- : 41
PtPLD6 : MGPRQPWHDLHCKIEGPAAYDVLTNFEQRWRKASKWSEFGR- : 41
VvPLD11 : KGPRQPWHDLHCKIEGPAAYDVLTNFEQRWRKATKWSEFGR- : 41
PtPLD17 : KAPRQPWHDLHCKIDGPAAYDVLINFEQRWRKATKWTELGL- : 41
PtPLD15 : KAPRQPWHDLHCKIDGPAAYDALINFEQRWRKATKWTELGL- : 41
AtPLDδ : KAPRQPWHDLHCRIDGPAAYDVLINFEQRWRKATRWKEFSL- : 41
OsPLDδ1 : GGPRQPWHDLHCRIDGPAAYDVLKNFEQRWRKATKWRERFR- : 41
PtPLD5 : KSPRQPWHDLHCKIEGPAAYDILTNFEQRWKKATKWRRIKK- : 41
VvPLD9 : RGPRQPWHDLHCKVEGPAAYDIMTNFEQRWRKAAKWRDFRL- : 41
OsPLDδ2 : YGPRQPWHDLHCKIEGPAAFDILTNFEQRWRKATKWKVNLK- : 41
OsPLDδ3 : SGPRQPWHDLHCKIEGPAAYDILTNFEQRWRKSAKWKVSVR- : 41
PtPLD10 : NCPREPWHDLHSRIDGPAAYDVLTNFEERWMKAAKPKGLKK- : 41
PtPLD4 : NCQREPWHDLHSRIDGPAAYDVLTNFEDRWMKAAKPKGLRK- : 41
AtPLDβ1 : GCPREPWHDLHSKIDGPAAYDVLTNFEERWLKAAKPSGIKK- : 41
AtPLDβ2 : GCPREPWHDLHSKIDGPAAYDVLTNFEERWLKAAKPHRINK- : 41
VvPLD10 : GCPREPWHDMHCKIDGPAAYDVLTNFQERWLKAAKPHGIKK- : 41
OsPLDβ2 : QGPREPWHDLHSKIDGPAAYDVLTNFEERWLKASKRSGVKK- : 41
OsPLDβ1 : RGPREPWHDLHSKIDGPAAYDVLQNFQERWLKASKRHGIKK- : 41
VvPLD1 : GCPREPWHDMHCRIDGPAAYDILTNFEERWLKASKPRGLQK- : 41
PtPLD1 : GCPRQPWHDLHCQIDGPAAYDILTNFEERWLKASKPRGMQK- : 41
AtPLDγ1 : DGPREPWHDLHSKIDGPAAYDVLANFEERWMKASKPRGIGK- : 41
AtPLDγ2 : VGPREPWHDLHSKIDGPAAYDVLANFEERWM-ASKPRGIGKG : 41
AtPLDγ3 : DGPREPWHDLHSKIDGPAAYDVLANFEERWMKASKPRGIGR- : 41
PtPLD2 : GGPREPWHDIHSRLEGPIAWDVLFNFEQRWKKQG-GKDLLVQ : 41
PtPLD13 : GGPREPWHDIHSRLEGPIAWDVLFNFEQRWKKQG-GKDLLVQ : 41
OsPLDα1 : GGPREPWHDIHSRLEGPIAWDVLYNFEQRWRKQG-GKDLLLQ : 41
VvPLD6 : GGPREPWHDIHSRLEGPIAWDVLFNFEQRWRKQG-GKDILLQ : 41
AtPLDα2 : GGPREPWHDIHCRLEGPIAWDVLYNFEQRWSRQG-GKDILVK : 41
AtPLDα1 : GGPREPWHDIHSRLEGPIAWDVMYNFEQRWSKQG-GKDILVK : 41
VvPLD7 : GGPREPWHDIHCRLEGAIAWDVLFNFEQRWRKQG-GKDLLVQ : 41
VvPLD8 : GGPRQPWHDVHCRLEGAIAWDVLFNFEQRWRKQG-GKDLLVQ : 41
PtPLD12 : GGPREPWHDVHCRLEGSIAWDVLVNFEQRWRKQG-KEDLLLQ : 41
OsPLDα2 : GGPREPWHDIHCRLEGPVAWDVLYNFEQRWRKQG-GKDLLVQ : 41
VvPLD2 : GGPREPWHDIHCRLEGAVAWDVLYNFEQRWRKQV-GEDVRPS : 41
OsPLDα5 : GGPREPWHDIHSKLEGPIAWDVLYNFEQRWRKQS-GHADLLV : 41
OsPLDα4 : GGPREPWHDIHSRLEGPVAWDVLYNFEQRWRKQS-GHGDLLV : 41
OsPLDα3 : GGPREPWHDIHSKIEGPAAWDVLYNFEQRWRKQG-GDKDLLL : 41
PtPLD7 : GGPREPWHDIHCKLEGPVAWDVLYNFEQRWTKQV-GDKLLIS : 41
AtPLDα3 : GGPREPWHDIHCKLDGPAAWDVLYNFEQRWMKQGSGRRYLI- : 41
OsPLDα6 : GGPREPWHDIHCRVEGPAAWDVLDNFEQRWRKQA-GRGKDSL : 41
OsPLDα7 : GGPREPWHDVHCRIEGPAAWDVLDNFEQRWRGQG-GAGGEAL : 41
OsPLDα8 : GGPREPWHDVHCRLEGRAAWDVLANFEQRWRKQA-PPEMAGC : 41
VvPLD5 : GGPREPWHDAHACITGEAARDVLTNFEQRWSKQC-NPSLLVP : 41
PtPLD14 : GGPREPWHDAHACIVGQAALDVLTNFEQRWNKQC-DGSVLVP : 41
AtPLDε : GGPREPWHDCHVSVVGGAAWDVLKNFEQRWTKQC-NPSVLVN : 41
PXPH
VvPLD4 : KYPRMPWHDVHCALWGPPCRDVARHFVQRWNYAKRNKAPNE- : 41
AtPLDζ2 : KYPRMPWHDVHCALWGPPCRDVARHFVQRWNHSKRNKAPNE- : 41
PtPLD8 : KYPRMPWHDVHCALWGPPCRDVARHFVQRWNFAKRNKAPYE- : 41
AtPLDζ1 : KHPRMPWHDVHCALWGPPCRDVARHFVQRWNYAKRNKAPYE- : 41
PtPLD16 : KYPRMPWHDVHCALWGPPCRDVARHFVQRWNYAKRSKAPYE- : 41
OsPLDζ1 : KYPRMPWHDVQCALYGPPCRDVARHFVQRWNYAKRNKAPNE- : 41
OsPLDζ2 : KYPRMPWHDVQCALYGPACRDIARHFVQRWNYAKRNKAPNE- : 41
PtPLD9 : KYPRMPWHDVHCSLWGPPCRDIARHFVQRWNHAKRSKAPNE- : 41
pR PWHD h 6 Gp a D nF RW
