# Supplementary material for: Genome-wide and molecular evolution analyses of the phospholipase D gene family in Poplar and Grape
Source: BMC Plant Biol. 2010 Jun 18;10:117. doi: 10.1186/1471-2229-10-117 (PMC3095279; doi:10.1186/1471-2229-10-117)
Supplement: Additional file 10 — Phylogenetic trees of the HKD1 domain(A) and HKD2 domain(B) sequences, respectively. The clades marked with the same color in the two trees represent the same kind of subgroup. [file 1471-2229-10-117-S10.PDF]

```
                               14
                          *         20
C2    PtPLD10  : HKTMQMMYETIYKALVEVGLE : 21
      PtPLD1   : HKTMQMMYETIYKALVEVGLE : 21
      VvPLD10  : HKTMQMMYETIYKALVEVGLE : 21
      AtPLDβ1  : HKTIQMMYETIYKALVETGLE : 21
      PtPLD4   : HKTMQMMYETIYKALDEVGLE : 21
      AtPLDγ3  : HKTMQMMYQTIYKALVEVGLD : 21
      AtPLDγ1  : HKTMQMMYQTIYKALVEVGLD : 21
      AtPLDγ2  : HKTMQMMYQTIYKALLEVGLD : 21
      AtPLDβ2  : HKTMQMMYGTIYNALVEAGLE : 21
      OsPLDβ1  : HKTMQMMYETIYRALKEEGLD : 21
      VvPLD1   : HKTMQMMYEMVYKALQEVGLE : 21
      OsPLDβ2  : KKTMQMMYEVIHKALKEVGLD : 21
      PtPLD6   : GQTMQMMYEVIAKELKSMNLE : 21
      PtPLD3   : GQTMQMMYEVIANELKSMNLE : 21
      PtPLD15  : SQTMQAMYEKIAQELKSMNLV : 21
      PtPLD17  : SQTMQAMYEKIAQELKSMDLV : 21
      VvPLD11  : GQTMQMMYDIIAQELQSMQLE : 21
      AtPLDδ   : SQTMQMMYDVIAKELKAVQSD : 21
      OsPLDδ3  : GQTMSMMYKIVAEALQKEGLD : 21
      OsPLDδ2  : SQTMSMMYKIIADALQMQGLV : 21
      PtPLD5   : GQTMAMMYKIIAKELEKAGLS : 21
      VvPLD9   : GQTMSMMYQIIGQAIHKAGLS : 21
      AtPLDε   : RETMSMMYQIIGEAIWEVGDK : 21
      VvPLD5   : RETMAMMYRLIGEAIDENGGS : 21
      PtPLD14  : RQTMAMMYKLVGEALQESGEP : 21
      OsPLDα8  : RLTVEMMYGIVTKAIDDAGLR : 21
      AtPLDα3  : RRTMEMMYTDIIIALRKKGLD : 21
      PtPLD7   : RRTMDMMYSDITEALVKKGLN : 21
      VvPLD8   : KRTMEMMYNDIAQALHAKGLK : 21
      VvPLD7   : KRTMEMMYNDIAQALHSKGLQ : 21
      PtPLD12  : KRTMEMMYSDIAEALQDKGVE : 21
      OsPLDα1  : RRTMEMMYTDITEALQAKGIE : 21
      VvPLD2   : RRTMEMMYTDIVQALQARGIH : 21
      OsPLDα2  : RRTMEMMYTDIAHAIQAKGID : 21
      OsPLDα6  : RRTMEMMYRDVDAAIQAKGIR : 21
      OsPLDα4  : RRTMEMMYADIAGALKAKRMD : 21
      OsPLDα5  : RRTMEMMYYDIAVALKANHSD : 21
      OsPLDα3  : RRTMEMMYYDIAVALEAKRIN : 21
      OsPLDα7  : RRTMEMMYKDVAAALAARGST : 21
      VvPLD6   : RRTMEMMYKDIIKALADKGIE : 21
      AtPLDα1  : RRTMEMMYKDVIQALRAQGLE : 21
      PtPLD2   : RRTMDMMYKDVIQALRAKGLE : 21
      PtPLD13  : RRTLEMMYKDVIEALRAKGLE : 21
      AtPLDα2  : KRTMEMMYKDVIKALRENGLE : 21
PXPH  PtPLD8   : NRVLEALYQRIMRAFNDKKCF : 21
      PtPLD16  : NRVLEALYRRIMRAFNDKKCF : 21
      VvPLD4   : NRVLEVLYRRIMQAYNDKKCF : 21
      OsPLDζ2  : NRVLEALYRRILRAEKEKRCF : 21
      OsPLDζ1  : NRVLEALYRRILRAEREKKRF : 21
      AtPLDζ1  : NRVLEALYKRILRAHNEKKIF : 21
      AtPLDζ2  : NRVLEALYRRILKAHEENKCF : 21
      PtPLD9   : NRVLDAIYKRVIQAYKENKCF : 21
                   t6  6Y  6  a
```
