# Supplementary material for: Genome-wide and molecular evolution analyses of the phospholipase D gene family in Poplar and Grape
Source: BMC Plant Biol. 2010 Jun 18;10:117. doi: 10.1186/1471-2229-10-117 (PMC3095279; doi:10.1186/1471-2229-10-117)
Supplement: Additional file 12 — Alignment of sequences of MEME motif 3 in PLD genes in Arabidopsis, rice, Poplar and Grape. Black and gray shadings indicate identical and conserved amino acid residues present in more than 50% of the aligned sequences, respectively. The colour bar and number above the sequence alignment represent MEME motifs. The sites marked by red boxes represent the "IYIENQ[FY]F" motif. [file 1471-2229-10-117-S12.PDF]

Table S2. Functional divergence estimated from pairwise comparison between C2-PLDs and PXPH-PLDs

Comparison	θ^a	$SE^b(\theta)$	$LRT^c(\theta)$	Sig.	$Q_k{}^d > 0.8$	$Q_k > 0.9$
C2-PLDs Vs PXPH-PLDs	0.64	0.043	217.60	P<0.01	75	40

[a] θ is the coefficient of functional divergence.

[b] $SE(\theta)$, standard error.

[c] $LRT(\theta)$ is a likelihood ratio test.

[d] Q_k, posterior probability.
