# Supplementary material for: Genome-wide and molecular evolution analyses of the phospholipase D gene family in Poplar and Grape
Source: BMC Plant Biol. 2010 Jun 18;10:117. doi: 10.1186/1471-2229-10-117 (PMC3095279; doi:10.1186/1471-2229-10-117)
Supplement: Additional file 14 — Alignment of sequences of MEME motif 2 in PLD genes in Arabidopsis, rice, Poplar and Grape. Black and gray shadings indicate identical and conserved amino acid residues present in more than 50% of the aligned sequences, respectively. The colour bar and number above the sequence alignment represent MEME motifs. The sites marked by red boxes represent the DRY motif. [file 1471-2229-10-117-S14.PDF]

Table S3. Parameter estimations and likelihood ratio tests for the site models

PLD type	ω underM_0	2Δl M3 vs. M0 (df 3)	2Δl M2 vs. M1 (df 2)	2Δl M8 vs. M7 (df 2)	Parameters in M8	Positive site under M8
C2	0.1145	3430.48**	0.02	0	p0=0.99999 p1= 0.00001 p= 0.50907 q= 3.35261 w= 1.00000	Not found
PXPH	0.1144	599.58**	0	0.8	p0=0.99436 p1=0.00564 p= 0.47307 q= 2.93704 w= 1.33500	627 S
SP	0.1547	156.27**	3.62	13.04**	p0=0.92218 p1=0.07782 p= 0.41537 q= 2.48819 w= 8.13743	3 K 18 Q 23 N
