# Supplementary material for: Genome-wide and molecular evolution analyses of the phospholipase D gene family in Poplar and Grape
Source: BMC Plant Biol. 2010 Jun 18;10:117. doi: 10.1186/1471-2229-10-117 (PMC3095279; doi:10.1186/1471-2229-10-117)
Supplement: Additional file 11 — Alignment of sequences of MEME motif 4 in PLD genes in Arabidopsis, rice, Poplar and Grape. Black and gray shadings indicate identical and conserved amino acid residues present in more than 50% of the aligned sequences, respectively. The colour bar and number above the sequence alignment represent MEME motifs. [file 1471-2229-10-117-S11.PDF]

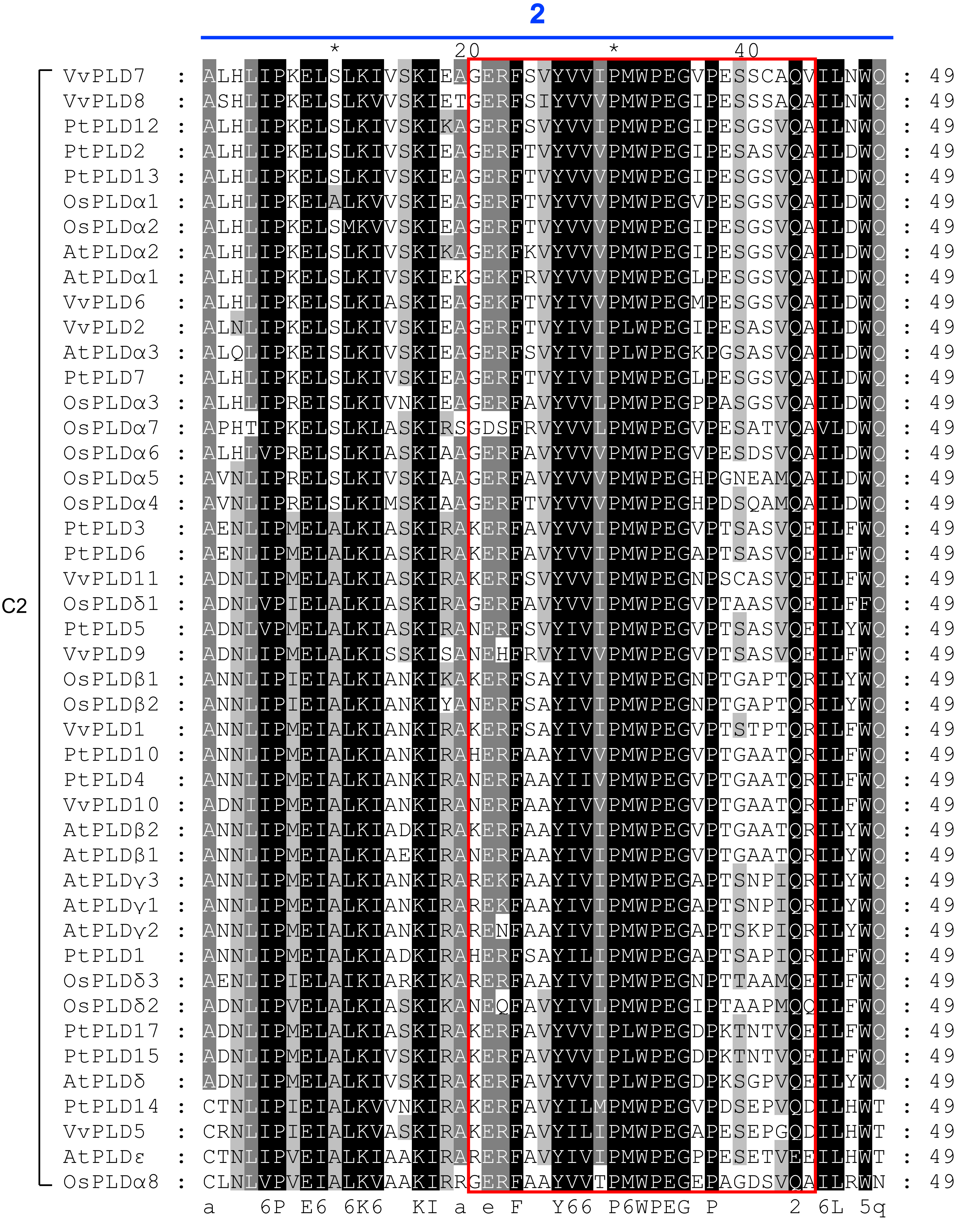
2
* 20 * 40
C2
VvPLD7 : ALHLIPKELSLKIVSKIEAGERFSVYVVIPMWPEGVPESSCAQVILNWQ : 49
VvPLD8 : ASHLIPKELSLKVVSKIETGERFSIYVVVPMWPEGIPESSSAQAILNWQ : 49
PtPLD12 : ALHLIPKELSLKIVSKIKAGERFSVYVVIPMWPEGIPESGSVQAILNWQ : 49
PtPLD2 : ALHLIPKELSLKIVSKIEAGERFTVYVVVPMWPEGIPESASVQAILDWQ : 49
PtPLD13 : ALHLIPKELSLKIVSKIEAGERFTVYVVVPMWPEGIPESGSVQAILDWQ : 49
OsPLDα1 : ALHLIPKELALKVVSKIEAGERFTVYVVVPMWPEGVPESGSVQAILDWQ : 49
OsPLDα2 : ALHLIPKELSMKVVSKIEAGERFTVYVVVPMWPEGIPESGSVQAILDWQ : 49
AtPLDα2 : ALHLIPKELSLKIVSKIKAGEKFKVYVVVPMWPEGIPESGSVQAILDWQ : 49
AtPLDα1 : ALHLIPKELSLKIVSKIEKGEKFRVYVVVPMWPEGLPESGSVQAILDWQ : 49
VvPLD6 : ALHLIPKELSLKIASKIEAGEKFTVYIVVPMWPEGMPESGSVQAILDWQ : 49
VvPLD2 : ALNLIPKELSLKIVSKIEAGERFTVYIVIPLWPEGIPESASVQAILDWQ : 49
AtPLDα3 : ALQLIPKEISLKIVSKIEAGERFSVYIVIPLWPEGKPGSASVQAILDWQ : 49
PtPLD7 : ALHLIPKELSLKIVSKIEAGERFTVYIVIPMWPEGLPESGSVQAILDWQ : 49
OsPLDα3 : ALHLIPREISLKIVNKIEAGERFAVYVVLPMWPEGPPASGSVQAILDWQ : 49
OsPLDα7 : APHTIPKELSLKLASKIRSGDSFRVYVVLPMWPEGVPESATVQAVLDWQ : 49
OsPLDα6 : ALHLVPRELSLKIASKIAAGERFAVYVVVPMWPEGVPESDSVQAILDWQ : 49
OsPLDα5 : AVNLIPRELSLKIVSKIAAGERFAVYVVVPMWPEGHPGNEAMQAILDWQ : 49
OsPLDα4 : AVNLIPRELSLKIMSKIAAGERFTVYVVVPMWPEGHPDSQAMQAILDWQ : 49
PtPLD3 : AENLIPMELALKIASKIRAKERFAVYVVIPMWPEGVPTSASVQEILFWQ : 49
PtPLD6 : AENLIPMELALKIASKIRAKERFAVYVVIPMWPEGAPTSASVQEILFWQ : 49
VvPLD11 : ADNLIPMELALKIASKIRAKERFSVYVVIPMWPEGNPSCASVQEILFWQ : 49
OsPLDδ1 : ADNLVPIELALKIASKIRAGERFAVYVVIPMWPEGVPTAASVQEILFFQ : 49
PtPLD5 : ADNLVPMELALKIASKIRANERFSVYIVIPMWPEGVPTSASVQEILYWQ : 49
VvPLD9 : ADNLIPMELALKISSKISANEHFRVYIVVPMWPEGVPTSASVQEILFWQ : 49
OsPLDβ1 : ANNLIPIEIALKIANKIKAKERFSAYIVIPMWPEGNPTGAPTQRILYWQ : 49
OsPLDβ2 : ANNLIPIEIALKIANKIYANERFSAYIVIPMWPEGNPTGAPTQRILYWQ : 49
VvPLD1 : ANNLIPMEIALKIANKIRAKERFSAYIVIPMWPEGVPTSTPTQRILFWQ : 49
PtPLD10 : ANNLIPMEIALKIANKIRAHERFAAYIVVPMWPEGVPTGAATQRILFWQ : 49
PtPLD4 : ANNLIPMEIALKIANKIRANERFAAYIIVPMWPEGVPTGAATQRILFWQ : 49
VvPLD10 : ADNIIPMEIALKIANKIRANERFAAYIVVPMWPEGVPTGAATQRILFWQ : 49
AtPLDβ2 : ANNLIPMEIALKIADKIRAKERFAAYIVIPMWPEGVPTGAATQRILYWQ : 49
AtPLDβ1 : ANNLIPMEIALKIAEKIRANERFAAYIVIPMWPEGVPTGAATQRILYWQ : 49
AtPLDγ3 : ANNLIPMEIALKIANKIRAREKFAAYIVIPMWPEGAPTSNPIQRILYWQ : 49
AtPLDγ1 : ANNLIPMEIALKIANKIRAREKFAAYIVIPMWPEGAPTSNPIQRILYWQ : 49
AtPLDγ2 : ANNLIPMEIALKIANKIRARENFAAYIVIPMWPEGAPTSKPIQRILYWQ : 49
PtPLD1 : ANNLIPMEIALKIADKIRAHERFSAYILIPMWPEGAPTSAPIQRILFWQ : 49
OsPLDδ3 : AENLIPIELAIKIARKIKARERFAAYIVIPMWPEGNPTTAAMQEILFWQ : 49
OsPLDδ2 : ADNLIPVELALKIASKIKANEQFAVYIVLPMWPEGIPTAAPMQQILFWQ : 49
PtPLD17 : ADNLIPMELALKIASKIRAKERFAVYVVIPLWPEGDPKTNTVQEILFWQ : 49
PtPLD15 : ADNLIPMELALKIVSKIRAKERFAVYVVIPLWPEGDPKTNTVQEILFWQ : 49
AtPLDδ : ADNLIPMELALKIVSKIRAKERFAVYVVIPLWPEGDPKSGPVQEILYWQ : 49
PtPLD14 : CTNLIPIEIALKVVNKIRAKERFAVYILMPMWPEGVPDSEPVQDILHWT : 49
VvPLD5 : CRNLIPIEIALKVASKIRAKERFAVYILIPMWPEGAPESEPGQDILHWT : 49
AtPLDε : CTNLIPVEIALKIAAKIRARERFAVYIVIPMWPEGPPESETVEEILHWT : 49
OsPLDα8 : CLNLVPVEIALKVAAKIRRGERFAAYVVTPMWPEGEPAGDSVQAILRWN : 49
a 6P E6 6K6 KI a e F Y66 P6WPEG P 2 6L 5q
